# MAPKAPK2 plays a crucial role in the progression of head and neck squamous cell carcinoma by regulating transcript stability

**DOI:** 10.1186/s13046-019-1167-2

**Published:** 2019-04-25

**Authors:** Sourabh Soni, Munish Kumar Saroch, Bal Chander, Narendra Vijay Tirpude, Yogendra S. Padwad

**Affiliations:** 1 Pharmacology and Toxicology Laboratory, Food and Nutraceuticals Division, CSIR-Institute of Himalayan Bioresource Technology (CSIR-IHBT), Palampur, H.P. India; 2 Department of Otorhinolaryngology, Head and Neck Surgery, Dr. Rajendra Prasad Government Medical College and Hospital (RPGMCH), Kangra, H.P. India; 3 Department of Pathology, Dr. Rajendra Prasad Government Medical College and Hospital (RPGMCH), Kangra, H.P. India; 4 Academy of Scientific and Innovative Research, Chennai, Tamil Nadu India

**Keywords:** HNSCC, Pathogenesis, MAPKAPK2, Transcript stability, RBPs, Xenograft

## Abstract

**Background:**

Head and neck squamous-cell carcinoma (HNSCC) ranks sixth among cancers worldwide. Though several molecular mechanisms of tumor initiation and progression of HNSCC are known, others remain unclear. Significance of p38/MAPKAPK2 (Mitogen-activated protein kinase-activated protein kinase-2) pathway in cell stress and inflammation is well established and its role in tumor development is being widely studied.

**Methods:**

We have elucidated the role of MAPKAPK2 (MK2) in HNSCC pathogenesis using clinical tissue samples, MK2-knockdown (MK2_KD_) cells and heterotropic xenograft mice model.

**Results:**

In patient-derived tissue samples, we observed that MK2 is reproducibly overexpressed. Increased stability of cyclin-dependent kinase inhibitor 1B (p27), mitogen-activated protein kinase phosphatase-1 (MKP-1) transcripts and decreased half-life of tumor necrosis factor-alpha (TNF-α) and vascular endothelial growth factor (VEGF) transcripts in MK2_KD_ cells suggests that MK2 regulates their transcript stability. In vivo xenograft experiments established that knockdown of MK2 attenuates course of tumor progression in immunocompromised mice.

**Conclusion:**

Altogether, MK2 is responsible for regulating the transcript stability and is functionally important to modulate HNSCC pathogenesis.

**Electronic supplementary material:**

The online version of this article (10.1186/s13046-019-1167-2) contains supplementary material, which is available to authorized users.

## Background

Globally, head and neck squamous cell carcinoma (HNSCC) having an estimated annual burden of 633,000 new cases and 355,000 deaths is the sixth most common cancer with the male to female ratio ranging from 2:1 to 4:1 [1]. Majority of head and neck cancers are HNSCCs (~ 90%) which pertain to malignancies in multiple anatomic subsites like oral cavity, oropharynx, hypopharynx, larynx and nasopharynx [2]. In India, 77,000 cases of HNSCCs are diagnosed every year, making it the second most common cancer in the subcontinent with various environmental and lifestyle risk factors as the primary causes [3]. The treatment for early-stage HNSCC is either single modality or employing various combinations of surgery, radiation and chemotherapy based on stage and primary site of the tumor [4]. Despite advances in surgical and other conventional treatment strategies in recent years, HNSCC continues to have a dismal prognosis with 30–47% recurrence rate as well as quite low 5-year survival rate among all cancers [5].

Systemic side effects like hepatic and cardiac toxicity as well as central nervous system disorders caused by the small molecules-based p38 inhibitors have hindered their translational use. This might be attributed to the fact that p38 regulates more than sixty substrates and therefore its direct inhibitors have failed in their clinical utility due to undesired side effects [6]. This has prompted researchers to look for novel therapeutic targets in downstream regulators of this signaling pathway, prominent among them being Mitogen-activated protein kinase-activated protein kinase-2 (MAPKAPK2 or MK2).

MK2 the downstream substrate of p38 mitogen-activated protein kinase (MAPK) governs the activation and deactivation of RNA binding proteins (RBPs) [7]. RBPs modulate the gene expression of mRNAs encoding several proto-oncogenes, cytokines, chemokines and pro-inflammatory factors that control cell-cycle progression, proliferation, angiogenesis, metastasis and cell death [8]. p38/MK2 signaling pathway has been implicated for its involvement in cell-cycle regulation, cell migration and inflammation [9]. Experimental evidences indicate that MK2, the prime target of p38, regulates the stability of essential genes involved in tumor pathogenesis that harbor adenine/uridine-rich elements (AREs) in their 3′-untranslated regions (3′-UTRs) [10]. It has been established that MK2 plays a significant role in a variety of cellular processes like cytoskeleton reorganization, chromatin remodeling, cell-cycle regulation and cell migration as indicated by its downstream substrates [7].

In this study, we observed overexpression and activation of MK2 in human HNSCC tissues as well as cell lines. Further, we investigated the expression levels of selected genes in clinical tissue samples harboring binding sites for MK2-regulated RBPs in their 3′-UTR and regulating HNSCC pathogenesis. We established that MK2 knockdown (MK2_KD_) in normoxia stabilized cyclin-dependent kinase inhibitor 1B (p27) but destabilized tumor necrosis factor-alpha (TNF-α) and vascular endothelial growth factor (VEGF) transcripts. Furthermore, we found that MK2_KD_ in tumor milieu mimicking hypoxic conditions stabilized p27 and mitogen-activated protein kinase phosphatase-1 (MKP-1) but destabilized TNF-α. The in vitro findings were further validated in vivo in a xenograft non-obese diabetic/severe combined immunodeficiency (NOD/SCID) mice model. Taken together, our findings show for the very first time that MK2 is responsible for regulating the transcript stability and is functionally important to modulate HNSCC pathogenesis.

## Methods

### Clinical tissue samples

HNSCC tissue samples along with adjacent normal samples (*n* = 100) were surgically obtained from patients in Department of Otorhinolaryngology, Head and Neck Surgery, RPGMCH, Kangra, India after appropriate prior informed written consent of the patients. The samples were not checked for Human papillomavirus infection. Similarly, the formalin-fixed and paraffin-embedded (FFPE) human HNSCC and normal tissue blocks (*n* = 50) were obtained from Department of Pathology, RPGMCH (Additional file 1: Table S1 contains patient details). Patient’s identity was kept anonymous throughout, and the study was approved by the Institutional Ethics Committee (IEC) of CSIR-IHBT, Palampur, India (Approval No. IEC/IHBTP-3/Jan.2014).

### Tissue pathology and immunohistochemistry

Collected samples and tissue blocks belonging to various subsites of the head and neck region (*n* = 50) were cut into 5 μm sections using microtomy and mounted on normal and lysine-coated glass slides for hematoxylin and eosin (H&E) and immunohistochemistry (IHC) staining, respectively. For H&E staining, the sections obtained on glass slides were deparaffinized, rehydrated and then stained with hematoxylin dye followed by eosin counterstaining. Standard reagents and protocols were used for H&E staining. The levels of expression and activation status of specific proteins (listed in Additional file 1: Table S2) in collected clinical samples were analyzed using IHC staining (protocol detailed in Additional file 1). Sections were then analyzed and imaged by a pathologist for cellular changes relating to HNSCC pathogenesis using bright field microscope (Leica DM 3000 with Leica application suite V4 image capture software).

### Antibodies

Primary antibodies raised against p38, p-p38, MK2, p-MK2, CCAAT/enhancer-binding protein delta (CEBPδ), p-CEBPδ, AU-rich element binding factor-1 (AUF1), p-AUF1, human antigen R (HuR), p-HuR, CUG triplet repeat RNA binding protein-1 (CUGBP1), tristetraprolin (TTP), and hypoxia-inducible factor-1 alpha (HIF-1α) were used in both IHC and Western blotting (WB) analysis of the collected clinical samples (Additional file 1: Table S2). Secondary antibodies used for WB were Anti-Mouse IgG-horseradish peroxidase (HRP) (Bio-Rad) and Anti-Rabbit IgG-HRP (Bio-Rad) raised in goat (1:3000 dilution).

### Cell lines and cell culture

Human HNSCC (FaDu, A-253, CAL27) and normal cell lines (HEPM, Hs680.Tr) were acquired from American Type Culture Collection (ATCC), USA. The cells were cultured at 37 °C, 5% CO2 in specific growth medium (Eagle’s Minimum Essential Medium (EMEM) for FaDu and HEPM; Dulbecco’s Modified Eagle Media (DMEM) for CAL27 and Hs680.Tr; McCoy’s5a modified medium for A-253 procured from Invitrogen) supplemented with 10% fetal bovine serum (FBS) and 1% antibiotic-antimycotic solution (Invitrogen). All the cell lines were properly quarantined and analyzed through monitoring of cell morphology and growth morphology under phase contrast before the start of any experimentation. We further analyzed the population doubling time and found the cell lines free of contamination as assessed by MycoFluor™ Mycoplasma Detection Kit (Invitrogen) and Cell Culture Contamination Detection Kit (Invitrogen) at the time of their use in experiments. All the procured cell lines were pre-authenticated from ATCC and used within 6 months of receipt for all the experimental work. For hypoxia exposure, cells plated in petriplates were incubated for 48 h in 0.5% O_2_ at 37 °C maintained in a hypoxia chamber (Bactrox, Shel-Lab). After 24 and 48 h, these hypoxia exposed cells were subjected to gene and protein expression analysis to validate the generation of hypoxia.

### Western blotting

For protein expression analysis, clinical tissue samples (*n* = 20)/cultured cells were lysed in protein lysis buffer followed by resolving on acrylamide gel and transfer onto membrane using standard protocol detailed in Additional file 1 [11]. Bands of specific proteins were detected and visualized using Clarity™ Western enhanced chemiluminescence (ECL) Substrate (Bio-Rad) with ECL imager (Azure). For quantification of protein bands ImageJ 1.49v software (NIH, USA) was used and statistical analysis was performed by one-way ANOVA using GraphPad Prism 7 software version 7.00.

### Sulforhodamine B assay

Sulforhodamine B (SRB) colorimetric assay was performed for cytotoxicity analysis of Actinomycin D (ActD) following standard protocol [12]. CAL27 cells were exposed to different concentrations of ActD (0.5, 1, 2.5, 5 and 10 μM) for 24, 48 and 72 h to evaluate its cytotoxicity. The experimental procedure has been detailed in Additional file 1.

### Transfection of CAL27 cells and stable shRNA knockdown experiments

CAL27 cells at about 60% confluence were transfected with the aid of Attractene reagent (Qiagen) with psi-U6.1 vectors expressing different 19-mer MK2-specific short hairpin RNA (shRNA) constructs (Additional file 1: Figure S1 and Table S3). Further, a non-specific scrambled control shRNA in psi-U6 vector (GeneCopoeia) was used, and the transfection was performed as per manufacturer recommended protocol detailed in Additional file 1.

Before conducting assays, the transfected cells were selected (1 μg/ml puromycin) to obtain stable transfectants and were further allowed to grow for atleast two generations. The confirmation of transfection was performed by imaging the green fluorescent protein (GFP) reporter expression inside the cells. The cells which were stably expressing shRNAs with an almost negligible expression of MK2 were called as MK2_KD_ cells. Selected cells were further grown and MK2_KD_ was confirmed by quantitative real time-PCR (qRT-PCR) and WB.

### qRT-PCR and determination of mRNA stability

Total RNA was extracted from collected surgical samples (*n* = 30 each for tumor and normal taking 5 samples each from six different head and neck subsites) and cell lines using RNeasy mini kit (Qiagen) following manufacturer’s protocol (detailed in Additional file 1). Extracted RNA was quantified by spectrophotometric measurement using Nanodrop (Thermo Fisher Scientific) before qRT-PCR analysis using Verso One-Step SYBR qRT-PCR kit, (Invitrogen) according to manufacturer recommended protocol (detailed in Additional file 1). Primers used for all the selected human genes were custom synthesized and obtained from Integrated DNA Technologies (Additional file 1: Table S4) while TaqMan probes and primers from Applied Biosystems (Additional file 1: Table S5). GAPDH was used as an endogeneous control for relative quantification of qRT-PCR data [13].

To evaluate the transcript stability, CAL27-MK2_KD_ cells along with non-transfected controls were treated with 1 μM of ActD (a sub-lethal dose). Total RNA was extracted after 0 min, 30 min, 1 h, 2 h, 4 h and 8 h of ActD treatment in both normoxic as well as hypoxic conditions. qRT-PCR was performed and the relative change in gene expression was evaluated to assess the mRNA stability of specific genes.

### Xenograft HNSCC mouse model

We developed a biologically relevant heterotropic xenograft model of HNSCC in immunocompromised mice. For this purpose male NOD/SCID mice of 6–8 weeks age were procured from the Experimental Animal Facility of Advanced Centre for Treatment, Research and Education in Cancer (ACTREC), Navi Mumbai, India. The animal study was approved by the Institutional Animal Ethics Committee (IAEC) of CSIR-IHBT, Palampur, India (Approval No. IAEC/IHBT-3/Mar 2017). The animals were housed in groups of four per individually ventilated cage (Tecniplast) under controlled conditions of 50 ± 10% humidity, 23 ± 2 °C temperature, and l2 h light/12 h dark cycle. The mice were randomly assigned into experimental or control groups and subjected to specific treatments according to the protocols (mice grouping has been detailed in Additional file 1: Table S6). For xenograft generation, one million cultured cells suspended in 100 μl of 1X PBS were injected subcutaneously in the right flank of the animals. Tumor growth and animal weights were regularly monitored. Seven weeks after graft inoculation, animals were sacrificed by CO_2_ inhalation and tumors were excised aseptically, weighed, used for RNA and protein extraction and processed for paraffin-fixation. Tumor sections were further analyzed using H&E and IHC staining.

### Statistical analysis

All the experimental procedures were conducted in triplicates unless indicated otherwise. The results presented here are expressed in the form of means±standard errors of the mean. Statistical significance between groups was analyzed by two-tailed, unpaired t-test using GraphPad Prism 7 software version 7.00. *p*-values< 0.05 were considered statistically significant.

## Results

### Patient characteristics and histopathological confirmation of HNSCC

In the present study, we obtained 100 HNSCC and adjoining normal clinical tissue samples (mean age 56.4; range 19–85 years) from patients comprising 75 males (mean age 58.5; range 19–79 years) and 25 females (mean age 49.7; range 26–85 years) and 50 FFPE-HNSCC and normal tissue blocks. The clinical samples used for the study originated from ~ 15 distinct subsites within the head and neck region, the majority belonging to the glottis/epiglottis, pharynx, tongue, nasal cavity and larynx. In males, the risk group (age group with the highest number of incidences) for HNSCC occurrence was 61–70 years with 26 cases while for females it was 41–50 years with 6 cases. Anamnesis of 34 random patients revealed history of smoking habit in case of 19 males (out of 23) and 1 female (out of 11) as well as alcohol consumption (10 males). Out of these 34 patients, 8 died in due course of time after surgery while 7 are living without any complication; however, no final information regarding survival status of other 19 patients was available after their successful completion of post-operative treatment. The detailed information regarding the patients is presented in Additional file 1: Table S1.

Histopathological evaluation of the clinical samples confirmed tumors comprising of squamous cells with moderate to severe nuclear polymorphism. Destruction of the basement membrane with the invasion of cells into the underlying submucosal to sub-epithelial region was observed in most of the cases (Additional file 1: Figure S2). Mucosal epithelial dysplastic change in certain sections suggested carcinoma in situ as previously postulated [14]. Keratin pearl formation was observed in most of the tumors. On the other hand, normal sections showed presence of stratified squamous epithelium with underlying intact basement membrane (Additional file 1: Figure S2).

### MK2 and its downstream target RBPs were found overexpressed and activated in HNSCC clinical tissue samples and cells

**Fig. 1:**
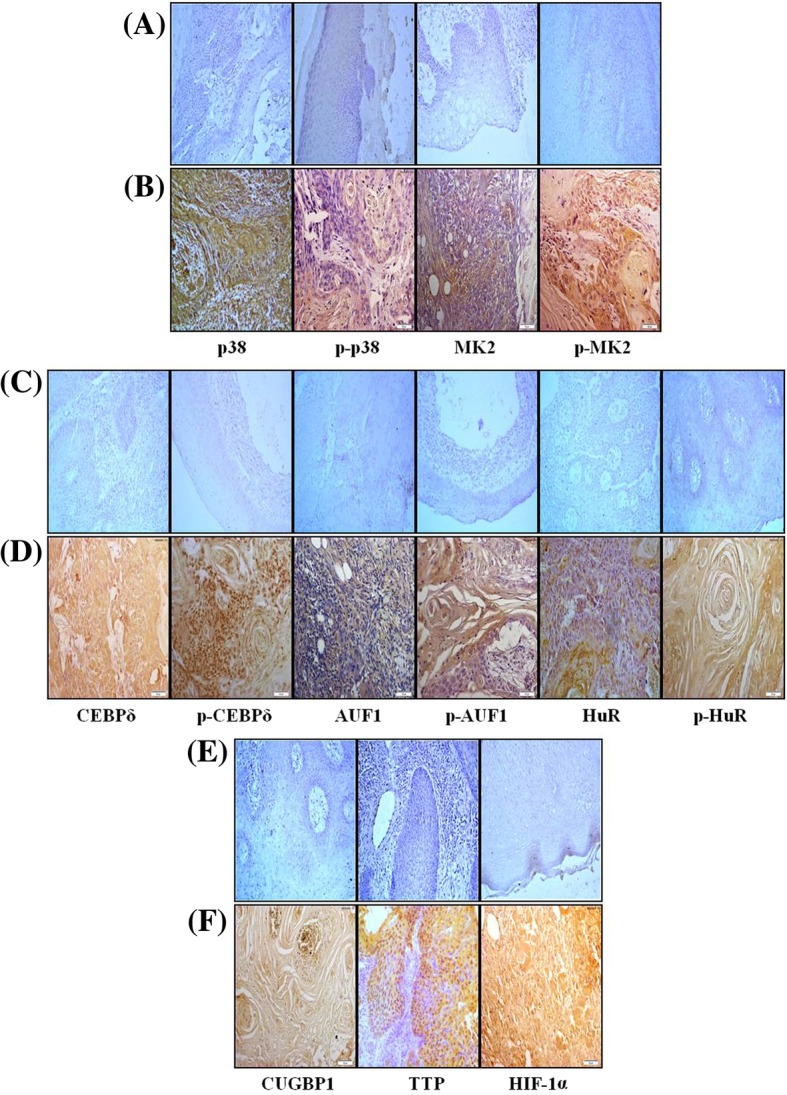
p38, MK2, RBPs and HIF-1α are overexpressed and activated in HNSCC. Representative IHC staining images of clinical tissue samples determining the expression and activation status of p38, p-p38, mitogen-activated protein kinase-activated protein kinase-2 (MK2), p-MK2, CCAAT/enhancer-binding protein delta (CEBPδ), p-CEBPδ, AU-rich element binding factor-1 (AUF1), p-AUF1, human antigen R (HuR), p-HuR, CUG triplet repeat RNA binding protein-1 (CUGBP1), tristetraprolin (TTP), and hypoxia-inducible factor-1 alpha (HIF-1α) in: (**a**, **c**, **e**) Normal tissue sections of head and neck region showing consistent negative staining in normal stratified squamous epithelium and, (**b**, **d**, **f**) HNSCC tissue sections showing consistent positive staining. The sections were subjected to IHC staining using specific primary antibodies followed by appropriate secondary antibody as described in Materials and Methods. Levels of expression of the above mentioned proteins were found to be relatively high in tumors (brown colour) as compared to normal controls. The images have been captured at 200x and the scale bar denotes 50 μm

The p38/MAPK pathway is widely implicated in invasion and metastasis of various tumors [15]. IHC staining confirmed that MK2 expression and phosphorylation is comparatively higher in most of the tumor samples (Fig. 1a and b). Similarly, the upstream factor p38/MAPK was also overexpressed and activated in tumor samples. Normal tissue sections of head and neck region showed consistent negative staining compared to their tumor counterparts (Fig. 1c-f). In an attempt to examine the interaction of MK2 with RBPs, we determined expression of MK2-regulated RBPs (CEBPδ, AUF1, HuR, CUGBP1, and TTP) using IHC and found their elevated expression and activation in tumor samples. Overexpression of hypoxia-inducible factor-1 alpha (HIF-1α) in tumor sections validated hypoxic conditions in the tumor core (Fig. 1c-f).

**Fig. 2:**
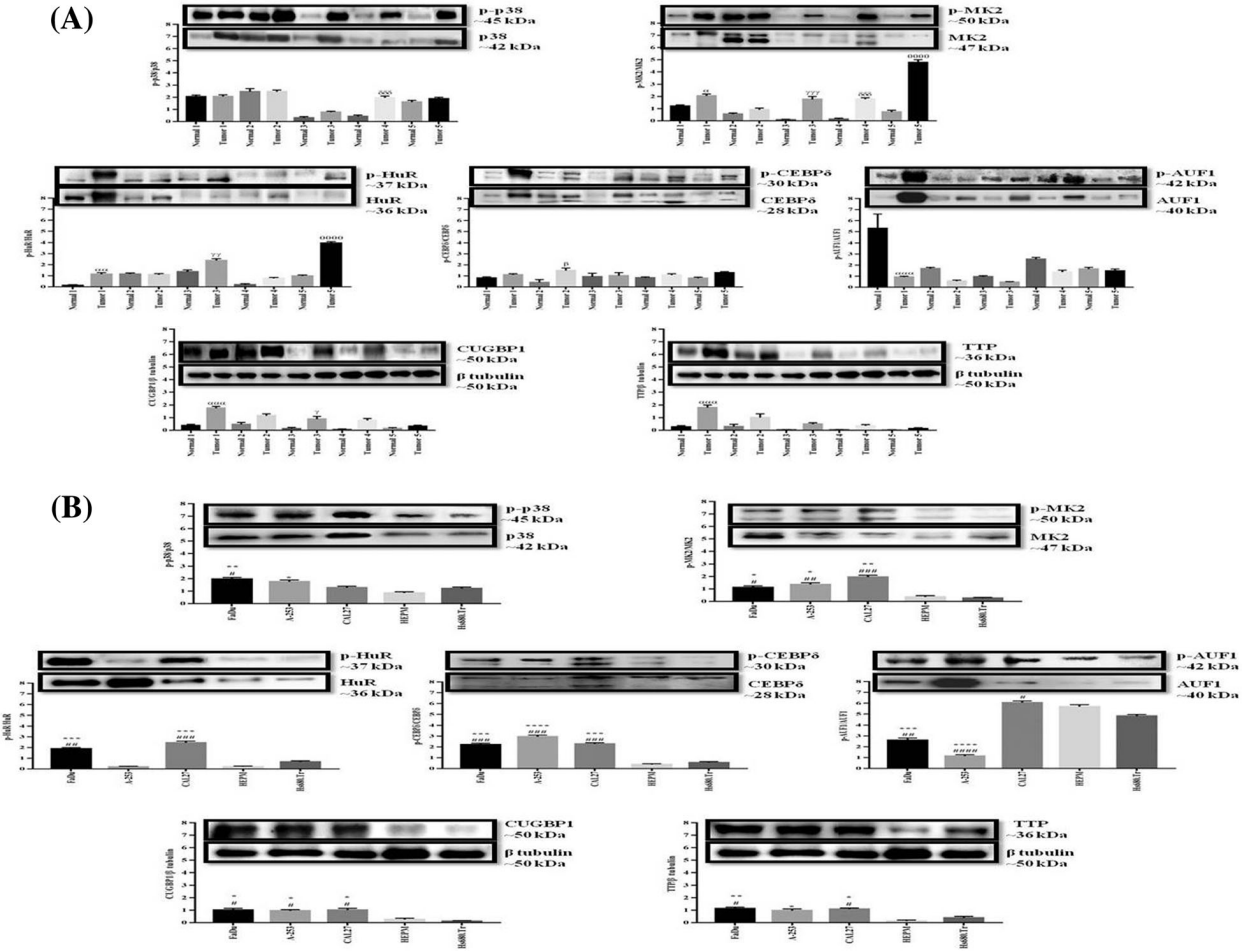
Western blot analysis confirmed higher levels of expression of specific proteins. Western blotting was performed to evaluate the levels of expression and activation status of p38, p-p38, MK2, p-MK2, HuR, p-HuR, CEBPδ, p-CEBPδ, AUF1, p-AUF1, CUGBP1 and TTP proteins in extracts prepared from: **a** Human clinical surgical samples and normal adjacent controls; **b** Human HNSCC cell lines (FaDu, A-253 and CAL27) and normal human cell lines of head and neck region (HEPM and Hs680.Tr). We observed higher expression levels and activation status of these proteins in tumor samples and HNSCC cells as compared with normal control samples and cell lines. β tubulin served as a loading control. The graphs represent change in protein expression/activation calculated as a ratio (arbitrary units). The results are expressed as means±standard errors of the mean, *n* = 3. α, *p* < 0.05; αα, *p* < 0.01; ααα, *p* < 0.001 represent the statistical significance of protein expression in tumor tissue 1 compared with normal control 1; and β, *p* < 0.05 represents the statistical significance of tumor tissue 2 compared with normal control 2; and γ, *p* < 0.05; γγ, *p* < 0.01; γγγ, *p* < 0.001 represent the statistical significance of tumor tissue 3 compared with normal control 3; and δδδ, *p* < 0.001 represent the statistical significance of tumor tissue 4 compared with normal control 4 and θθθθ, *p* < 0.0001 represent the statistical significance of tumor tissue 5 compared with normal control 5. Similarly, *, *p* < 0.05; **, *p* < 0.01; ***, *p* < 0.001 and ****, *p* < 0.0001 represent the statistical significance of protein expression in human HNSCC cell lines compared to HEPM while #, *p* < 0.05; ##, *p* < 0.01; ###, *p* < 0.001 and ####, *p* < 0.0001 represent the statistical significance of human HNSCC cell lines compared to Hs680.Tr

Next, we evaluated and then quantified the MK2 protein expression and activation in HNSCC and adjacent normal tissues using WB analysis. Activation status and overexpression of the target proteins has been calculated by normalizing them with their phospho-forms/β tubulin as shown in Fig. 2. Consistent with our histopathological and immunohistochemical findings, WB analysis also confirmed p38 and MK2 significantly overexpressed and activated in majority of tumor samples as compared to adjacent normal tissues (Fig. 2a). Similarly, RBPs were found to have consistent overexpression and activation of these proteins in clinical tumors as compared to adjacent normal tissues (AUF1 was the only exception with lower activation in tumor tissues) (Fig. 2a). These observations in clinical samples were validated and also quantified in vitro by performing WB analysis in human HNSCC (FaDu, ATCC HTB-43 pharynx squamous cell carcinoma; A-253, ATCC HTB-41 submaxillary salivary gland epidermoid carcinoma; CAL27, ATCC CRL-2095 tongue squamous cell carcinoma) and normal head and neck cell lines (HEPM, ATCC CRL-1486 palatal mesenchyme; Hs680.Tr, ATCC CRL-7422 trachea normal). In agreement to our earlier findings, we observed a significant increase in p38 and MK2 protein levels and their activation in HNSCC cells as compared to normal cell lines (Fig. 2b). As evident from our findings, CAL27 cells showing significantly high expression and activation of MK2 were selected for further experimentation. Furthermore, in consonance with our previous observations in tumor tissues we found that RBPs (with the same exception of AUF1) showed a higher level of expression and activation status in HNSCC cells respective to normal head and neck cells (Fig. 2b). β tubulin was used as a loading control in this study. In a nutshell, it is evident that the expression and activation of p38, MK2 and MK2-regulated RBPs is elevated compared with respective normal controls, hence, potentiating the hypothesis that MK2 regulates the pathogenesis of HNSCC via a probable interaction with RBPs.

### Changes in the expression levels of genes involved in the pathogenesis of HNSCC in clinical tissue samples

**Fig. 3:**
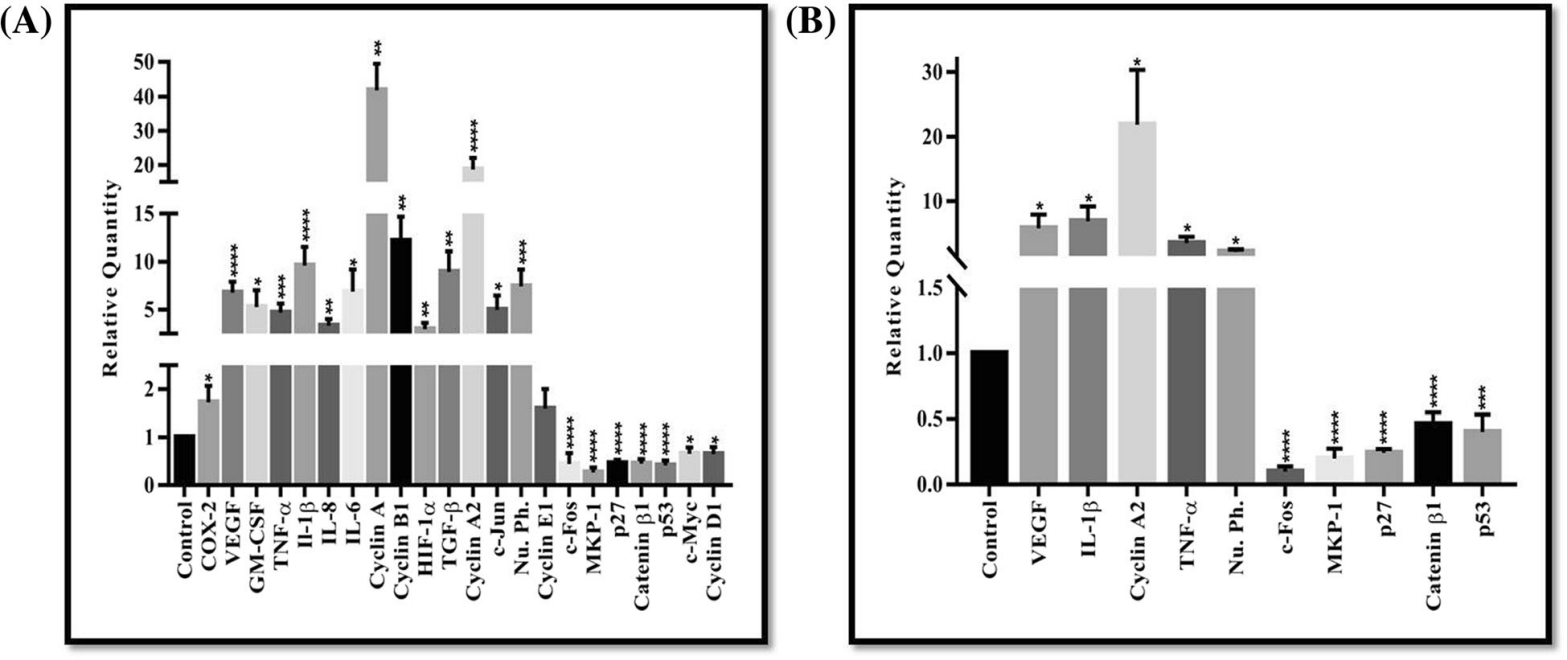
Relative gene expression levels of HNSCC pathogenesis-specific genes in clinical tissues. **a** Graphical representation of qRT-PCR results (using SYBR Green chemistry). **b** Graphical representation of qRT-PCR results (using TaqMan chemistry). The graphs show the relative fold change values/relative gene expression of various genes involved in HNSCC pathogenesis. Histograms represent the levels of up/down-regulation of a gene as compared to control samples. Relative gene expression was obtained after normalization with endogenous human GAPDH and determination of the difference in threshold cycle (C_t_) between tumor and normal tissues was performed using the 2^-ΔΔC^_t_ method. All the qRT-PCR assays were performed in triplicate. The results are expressed as means±standard errors of the mean. *, *p* < 0.05; **, *p* < 0.01; ***, *p* < 0.001 and ****, *p* < 0.0001 represent the statistical significance compared with control

To elucidate the role of MK2 in regulating the expression of key genes involved in HNSCC tumorigenesis, expression analysis of selected genes that are involved in a plethora of critical cellular processes like cell-cycle regulation, angiogenesis, metastasis and cell death was performed. These genes play crucial roles in HNSCC pathogenesis and harbors ARE sites in their 3′-UTR for the binding of specific MK2-regulated RBPs. For this, extracted total RNAs from the collected clinical samples were used as templates for the determination of RNA copy number by qRT-PCR of the target genes (listed in Additional file 1: Table S7). From qRT-PCR analysis using SYBR Green chemistry, it was evident that all of the 22 selected genes showed significant variations in the levels of expression with 15 genes overexpressing while 7 down-regulating in tumor tissues as compared to controls (*p* < 0.05). Cyclin A was the highest up-regulated gene with a relative fold change (R) of ~ 40 as compared to normal controls, while MKP-1 (R ≈ 0.2) was the most down-regulated gene (Fig. 3a and Additional file 1: Table S7). Additional file 1: Figure S3 is a graphical representation of the qRT-PCR results (SYBR Green chemistry) showing top five significantly up-regulated and down-regulated genes, respectively.

Further, qRT-PCR results obtained using SYBR Green chemistry were validated using more specific and reliable TaqMan chemistry for the top 10 significantly up/down-regulated genes (Fig. 3b and Additional file 1: Table S8). Our findings confirmed the validation for the 10 selected genes which were showing consistent expression levels as observed in SYBR Green chemsitry. Here, Cyclin A2 (R ≈ 22) was found to be the highest up-regulated gene in tumor samples, while c-Fos (R ≈ 0.1) was the most down-regulated gene (Fig. 3b and Additional file 1: Table S8). Statistical analysis affirmed that the levels of expression of genes in tumor tissues varied significantly relative to the normal samples (*p* < 0.05) (Fig. 3b). Additional file 1: Figure S4 is a graphical representation of the qRT-PCR (Taqman chemistry) results showing the up/down-regulated genes. Taken together, our results indicated that pro-inflammatory genes like VEGF, TNF-α are up-regulated while tumor suppressor genes like p27, MKP-1 are down-regulated in tumor samples as compared to normal controls. In brief, our findings suggested that expression levels of these genes varied significantly in HNSCC as compared to normal samples, thus, potentiating their crucial role in HNSCC pathogenesis.

### MK2 regulates the expression of important genes and plays a crucial role in HNSCC pathogenesis

**Fig. 4:**
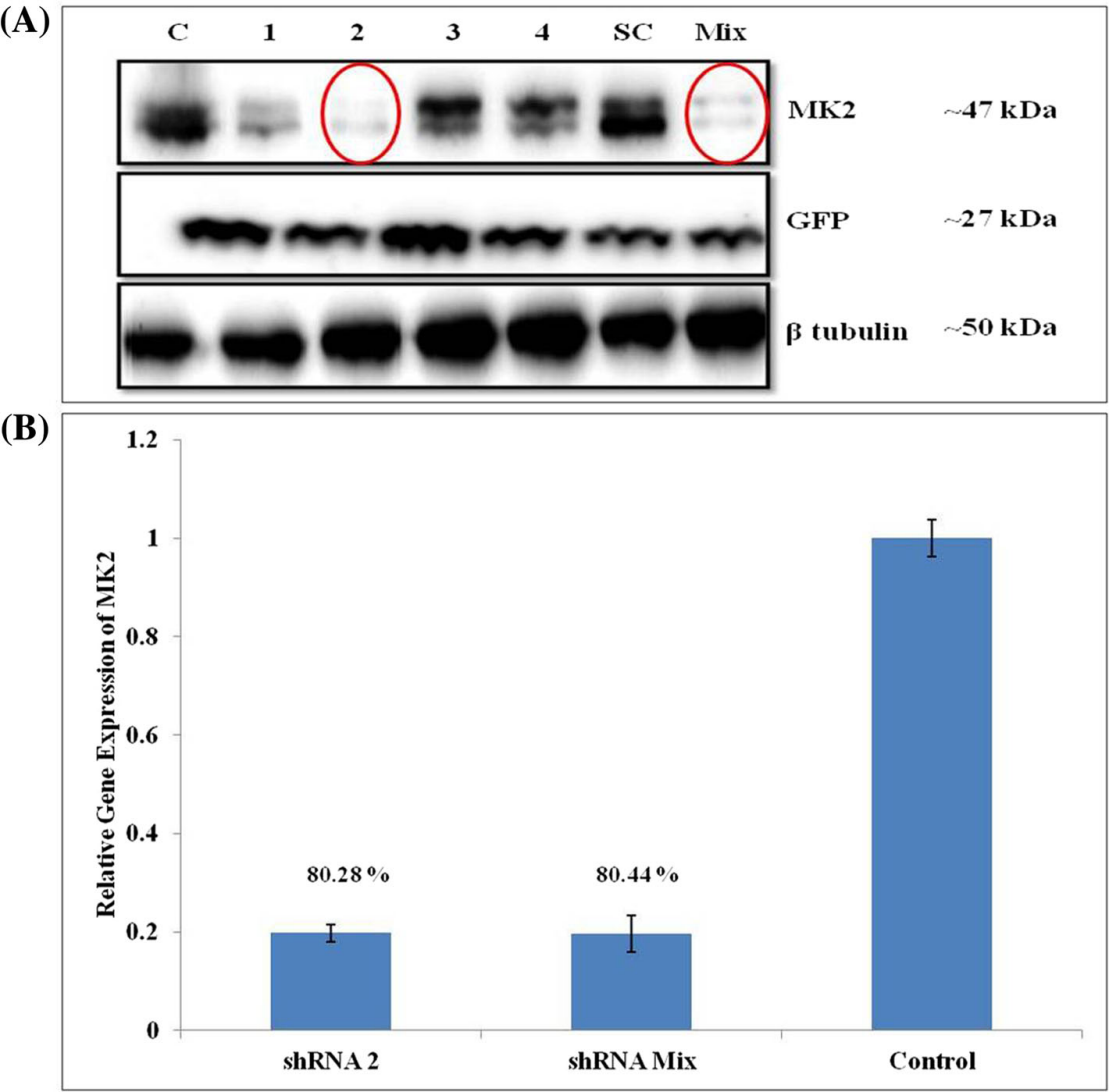
Validation of shRNA-GFP construct transfection into CAL27 cells and MK2 knockdown. **a** For the validation of MK2_KD_ the expression levels of MK2 were confirmed by Western blot analysis against non-transfected controls using MK2-specific antibody. Negligible expression in shRNA 2 and shRNA Mix transfected cells as compared to normal and scrambled controls confirmed that the protein expression of MK2 has been down-regulated in these. C=Non-transfected control; 1, 2, 3, 4, SC = shRNA-GFP construct 1, 2, 3, 4, Scrambled Control transfected, respectively; Mix = Co-transfected with shRNA-GFP constructs 1, 2, 3, 4. GFP served as an input control while β tubulin was used as a loading control in this case. **b** qRT-PCR analysis of the CAL27-MK2_KD_ cells established that the shRNA transfection lead to ~ 80% knockdown of MK2 compared to non-transfected control as shown in the histograms. All the qRT-PCR assays were performed atleast thrice. The results are expressed as means±standard errors of the mean

To ascertain the role of MK2 in regulation of gene expression involved in HNSCC pathogenesis as identified earlier, expression levels of MK2 in MK2_KD_ cells were confirmed by WB analysis against non-transfected controls using MK2-specific antibody. The confirmation of transfection was performed by imaging the GFP reporter expression inside the cells using immunofluorescence microscopy (Carl Zeiss), imaging flow cytometer (Amnis, Merck) and EVOS FL Auto 2 imaging system (Thermo Fisher Scientific) (Additional file 1: Figure S5A-C). Negligible levels of expression in shRNA 2 and Mix (combination of an equal quantity of shRNA 1, 2, 3 and 4) transfected cells as compared to normal and scrambled control confirmed that the protein expression of MK2 has been significantly suppressed, thereby, validating MK2_KD_ (Fig. 4a). GFP was used as an input control in this case. Furthermore, RNA was extracted from MK2_KD_ cells to evaluate the percentage of MK2_KD_ using qRT-PCR analysis. Our findings confirmed that compared to non-transfected control, the shRNA transfected cells showed ~ 80% MK2_KD_ (Fig. 4b).

Finally, the effect of MK2_KD_ on the transcript copy number of previously identified genes playing critical role in HNSCC pathogenesis was observed. As expected, a reversal in the levels of expression of previously validated genes was noted in MK2_KD_ cells using qRT-PCR analysis employing both SYBR Green and TaqMan chemistry (Additional file 1: Figure S6 and Table S9). This finding further affirmed the pivotal role of MK2 in regulating the expression of HNSCC pathogenesis-linked genes.

### MK2 regulates the transcript stability of TNF-α, VEGF, p27 and MKP-1 transcripts

To further identify the transcript regulatory characteristic of MK2, stability of the qRT-PCR validated transcripts was assessed in both normoxic as well as tumor microenvironment mimicking hypoxic conditions. qRT-PCR results postulated that after 48 h of hypoxia exposure CAL27 cells showed ~ 6 fold increase in the transcript levels of HIF-1α (a hypoxia indicator) (Additional file 1: Figure S7). WB further confirmed higher levels of HIF-1α protein expression as compared to normoxic cells (Additional file 1: Figure S8), thus validating the generation of hypoxia in the cultured cells which were further used in mRNA decay study in presence and absence of MK2.

CAL27 cells cultured under both normoxia and hypoxia were treated with different concentrations of ActD to assess its effect on the cells. Cytotoxicity evaluation through SRB assay established the cytotoxic potential of ActD (Additional file 1: Figure S9) and enabled us to choose a sub-lethal concentration of ActD for transcript stability experiment. To further support our hypothesis that MK2 is directly involved in the regulation of transcript stability of key genes involved in HNSCC pathogenesis, we evaluated the mRNA turnover of the ten previously validated transcripts in both normoxia as well as hypoxia exposed CAL27-MK2_KD_ cells. Cells were treated with ActD to block transcription, and then at different time points, the transcript levels were determined by qRT-PCR.

**Fig. 5:**
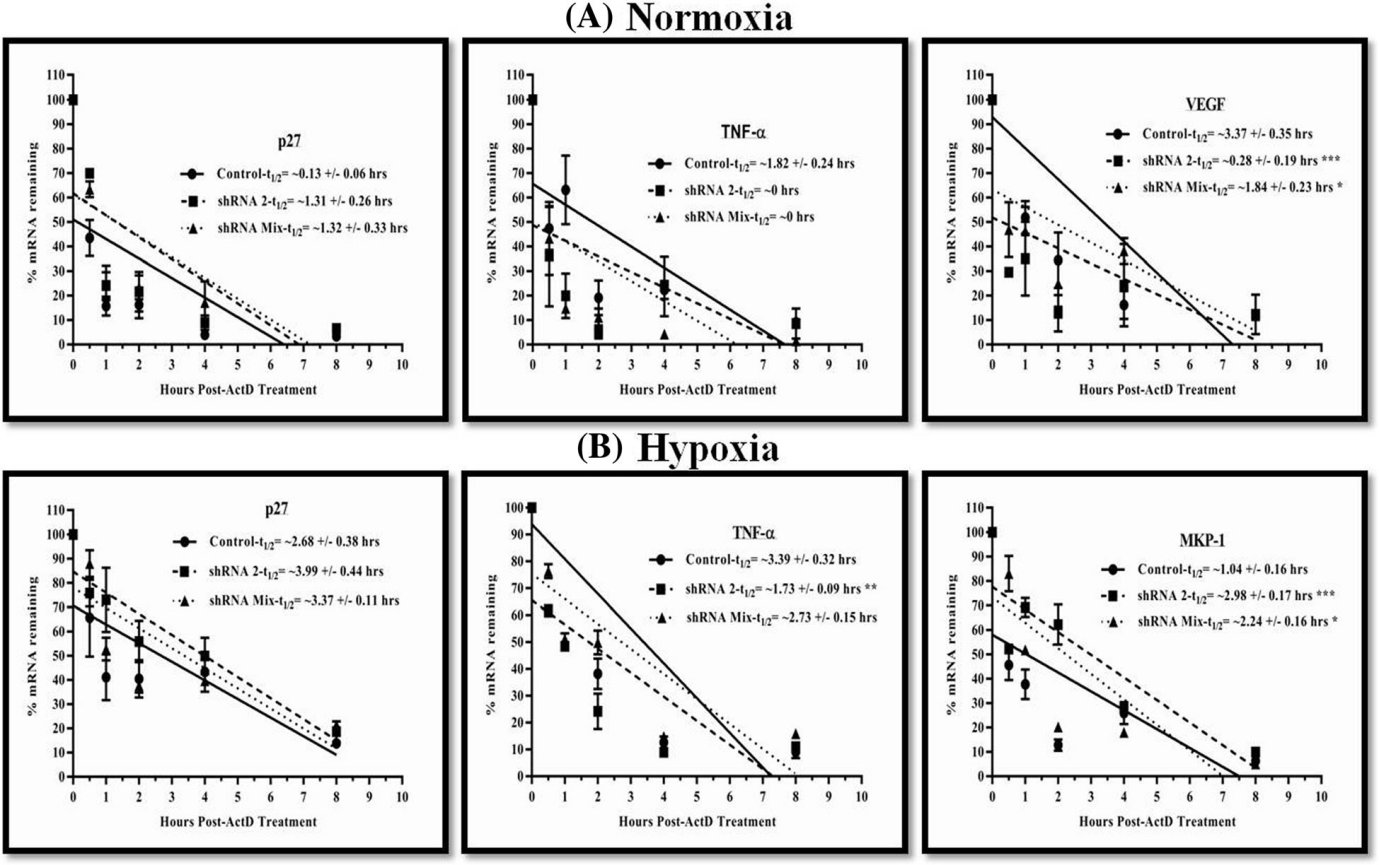
MK2 regulates the transcript stability of genes involved in HNSCC pathogenesis. CAL27-MK2_KD_ cells treated with ActD were analyzed for the transcript levels of various genes by performing qRT-PCR at different time points (0 min, 30 min, 1 h, 2 h, 4 h and 8 h post-ActD treatment). Results of the kinetic study analyzed using linear regression of mRNA decay rate have been graphically presented here. Following the transcriptional inhibition with ActD, qRT-PCR was performed and results obtained were used to plot the decay curves and half-lives of (**a**) p27, TNF-α and VEGF in normoxia (**b**) p27, TNF-α and MKP-1 in hypoxia. GAPDH serves as an endogeneous control. The graphs showcase that MK2_KD_ increased the stability of p27 and MKP-1 transcripts but decreased the half-life (t_1/2_) of TNF-α and VEGF transcripts in CAL27 cells. All the qRT-PCR assays were performed in triplicate. The results are expressed as means±standard errors of the mean. *, *p* < 0.05; **, *p* < 0.01 and ***, *p* < 0.001 represent the statistical significance compared with control

Results of the kinetic study analyzed using linear regression of mRNA decay rate established that MK2_KD_ increased the half-life (t_1/2_) of p27 and MKP-1 transcripts while an opposing effect was observed for TNF-α and VEGF transcripts in CAL27 cells. We observed that in normoxic conditions, t_1/2_ of p27 transcripts increased from ~ 0.13 to ~ 1.3 h while in hypoxia it showed an increase from ~ 2.7 to ~ 4 h (Fig. 5a and b). Similarly, hypoxia tends to stabilize MKP-1 transcripts by increasing t_1/2_ from ~ 1 to ~ 3 h. Our findings revealed that MK2_KD_ destabilized TNF-α transcripts with t_1/2_ decreasing from ~ 1.8 to ~ 0 h in normoxia and from ~ 3.4 to ~ 1.7 h in hypoxia. In normoxia, we observed a robust decay that could not be determined by our best-fit linear regression equation. Similarly, destabilization of VEGF transcripts occurred in normoxic conditions where t_1/2_ decreased from ~ 3.4 to ~ 0.3 h in CAL27-MK2_KD_ cells. Statistical analysis of t_1/2_ of the transcripts revealed that the destabilization of VEGF transcripts in normoxia was significant (*p* < 0.05). Similarly, in hypoxic conditions MKP-1 transcripts stabilization and the decay of TNF-α transcripts in CAL27-MK2_KD_ cells were significant (Fig. 5a and b). Cultured cells exposed to hypoxia confirmed the role of MK2 in post-transcriptional gene regulation in tumor microenvironment. In a nutshell, hypoxia stabilized p27 and MKP-1 transcripts while it caused the destabilization of TNF-α in CAL27-MK2_KD_ cells. Similarly, normoxia lead to the decay of TNF-α and VEGF transcripts, while at the same time stabilizing p27 in the absence of MK2. Taken together, our results indicated that MK2 directly controls the mRNA turnover at the post-transcriptional level by regulating the transcript stability.

### MK2_KD_ attenuates tumor progression in xenograft model

**Fig. 6:**
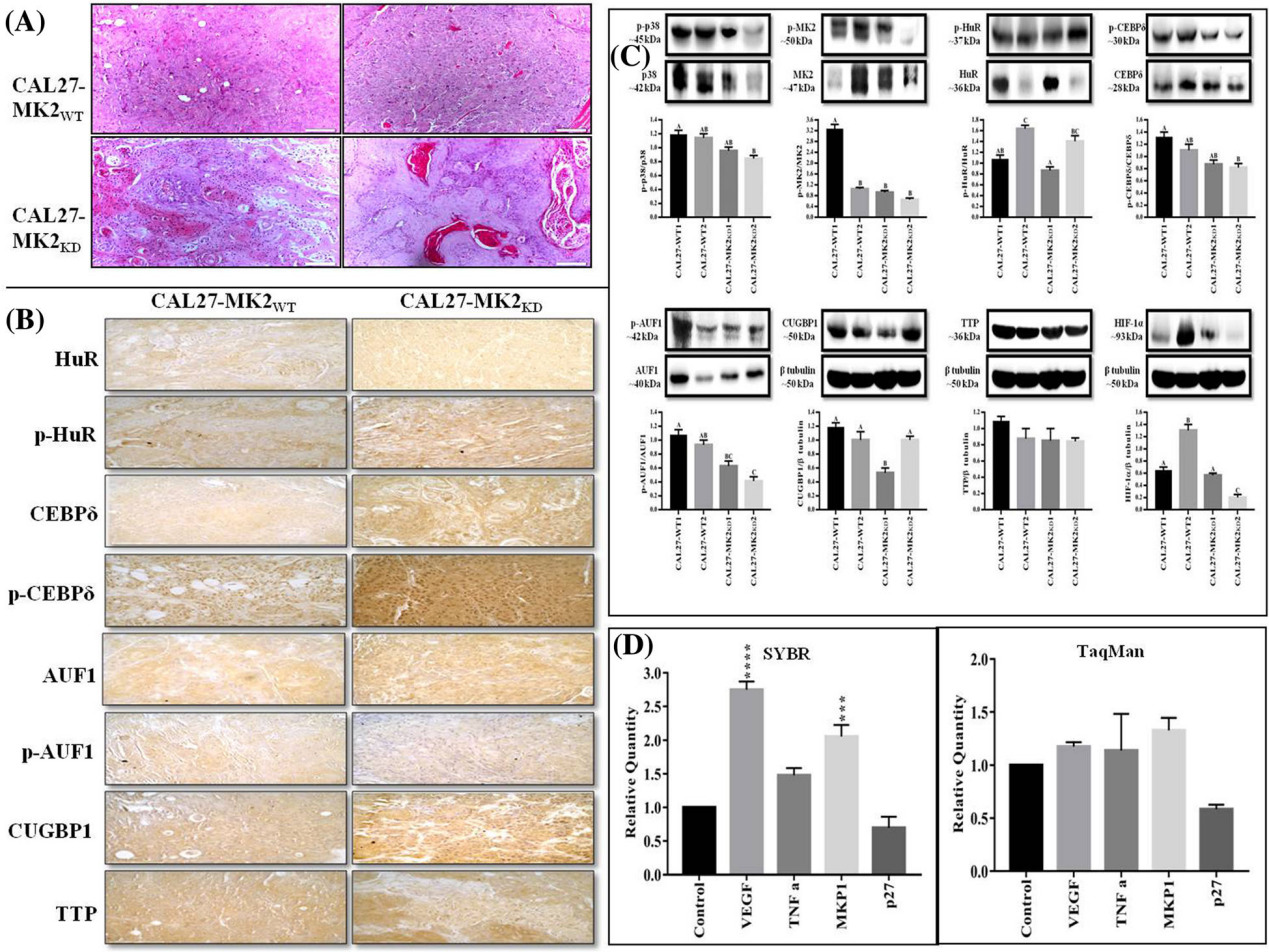
Xenograft establishes that MK2_KD_ attenuates tumor progression. **a** Histopathological examination revealed less differentiated and more aggressive tumors in CAL27-MK2_WT_ than CAL27-MK2_KD._ The images have been captured at 100x and the scale bar denotes 100 μm. **b** IHC showed expression and activation status of RBPs is more prevalent in CAL27-MK2_WT_ as compared to CAL27-MK2_KD_ group. The images have been captured at 880x (40x objective) and the scale bar denotes 100 μm. **c** Protein expression analysis using tumor lysates showed that the expression of p38, MK2 and RBPs is higher in CAL27-MK2_WT_ as compared to CAL27-MK2_KD._ β tubulin served as a loading control. The graphs represent change in protein expression/activation calculated as a ratio (arbitrary units). The results are expressed as means±standard errors of the mean, *n* = 3. Significant differences between CAL27-MK2_WT_ and CAL27-MK2_KD_ groups are indicated by different alphabets (*p* < 0.05). **d** qRT-PCR analysis showed that VEGF, TNF-α and MKP1 transcripts were upregulated while p27 was downregulated in CAL27-MK2_KD_ as compared to CAL27-MK2_KD_ tumors (as evaluated by SYBR and TaqMan chemistry). Relative gene expression was obtained after normalization with endogenous human GAPDH and determination of the difference in threshold cycle (C_t_) between CAL27-MK2_WT_ and CAL27-MK2_KD_ groups was performed using the 2^-ΔΔC^_t_ method. All the qRT-PCR assays were performed in triplicate. The results are expressed as means±standard errors of the mean. ***, *p* < 0.001 represent the statistical significance compared with control

In order to closely mimic the tumor microenvironment and validate our in vitro findings we generated a xenograft model in immunocompromised mice. Gross tumor growth was observed in all the animals grafted with CAL27-MK2 wild type (MK2_WT_) and CAL27-MK2_KD_ cells, suggesting successful xenograft generation. The tumors were well circumscribed, totally encapsulated and did not show metastasis to any other organ or interaction with surrounding tissues till our study period of seven weeks post-grafting. Tumors were moderately to well differentiated with mild microinvasion of capsules. The in vitro results were recapitulated in in vivo experiments. The xenografts showed slower and less aggressive tumor progression in CAL27-MK2_KD_ derived tumors as compared to CAL27-MK2_WT_ derived tumors suggesting that loss-of-MK2 attenuated tumor growth. Histopathological analysis suggested that tumors derived from CAL27-MK2_WT_ group were more aggressive and less differentiated (Fig. 6a). Further, IHC showed the expression and activation status of RBPs is more prevalent in CAL27-MK2_WT_ as compared to CAL27-MK2_KD_ group (Fig. 6b). Similarly, protein expression analysis using tumor lysates showed that the expression of p38, MK2 and RBPs is higher in CAL27-MK2_WT_ as compared to CAL27-MK2_KD_ group suggesting a prominent role of MK2 in HNSCC progression via RBP mediated gene regulation (Fig. 6c). Furthermore, qRT-PCR analysis showed that VEGF, TNF-α and MKP1 transcripts were upregulated while p27 was downregulated in tumors derived from CAL27-MK2_KD_ cells when compared with CAL27-MK2_WT_ tumors (Fig. 6d). These results are in consonance to our in vitro findings in CAL27-MK2_KD_ cells. Parameters like mice weight, tumor gross weight, biochemical and haematological analysis were also evaluated (Additional file 1: Table S10 and S11).

## Discussion

HNSCC accounts for 4.3% of all cancer cases globally and estimates project about half-million new cases worldwide annually, ranking HNSCC sixth among all cancers in incidences [16]. Post-transcriptional regulation of gene expression in tumor versus normal tissues is a highly unexplored area and is especially not well understood in HNSCC. Transcript processing is being increasingly recognized as the most important regulatory step of gene expression in mammals. It is believed that specific interactions between cis-acting structural elements (AREs) located in the 3′-UTRs of proto-oncogenes, growth factors, cytokines, transcription factors and other important proteins with trans-acting RBPs tend to change the protein translation landscape of stressed cells [10, 17].

p38/MAPK, a signal transducing enzyme present in all eukaryotes, is the prime regulatory hub where inflammation and stress responses are regulated [18]. It plays a major role in regulating MK2 expression in response to diverse stimuli and triggers elaborate biological signal transduction cascades allowing cells to interpret a wide range of external signals [19, 20]. MK2 activation generates a plethora of different biological effects targeting diverse cellular processes like cell-cycle progression, cytoskeletal architecture, transcript stability and protein translation via regulating the activation and deactivation cycles of RBPs [10]. Surprisingly, till date, the biological significance of MK2 in cancer is not well elucidated. A better understanding of the role of MK2 in tumor progression could provide new insights into the enigma of the post-transcriptional gene regulation in cancer.

To this end, our study was aimed to explore the role of MK2 in post-transcriptional control of crucial genes involved in HNSCC pathogenesis. Here, we demonstrate that MK2 plays an essential role in post-transcriptional gene expression in HNSCC by regulating the mRNA turnover. p38/MK2 signaling establishes a pivotal inflammatory axis with substantial reports affirming its critical role in stress responses [21, 22]. Recent reports of MK2 overexpression in tumors suggested that its oncogenic activity is required for the malignant growth [23, 24]. In consonance with these findings, we have identified that MK2 is consistently overexpressed in HNSCC and regulates transcript stability of genes involved in HNSCC progression.

RBPs like TTP, HuR, AUF1, CUGBP1 and CEBPδ can directly or indirectly control turnover of mRNAs encoding tumor pathogenesis-related factors. The aberrant expression of RBPs can alter the gene expression patterns and, subsequently, involve in carcinogenesis [25, 26]. The complex mechanisms of post-transcriptional regulation of cytokines via MK2-dependent phosphorylation of RBPs have been discussed in several excellent reviews [18, 20]. Here we have established significant overexpression of MK2 in tumor tissues and HNSCC cells. Further, it has been observed that MK2 is activating TTP, HuR, CUGBP1 and CEBPδ while deactivating AUF1. These activation and deactivation cycles of RBPs are further responsible to control the downstream genes in this pathway. In this report, we have also found significant up/down-regulation in transcript levels of crucial genes regulating HNSCC pathogenesis in clinical samples as compared to adjacent normal tissues. We also investigated the role of MK2 in modulating mRNA turnover of specific genes in HNSCC cells under hypoxic tumor microenvironment and normoxia. Hypoxia, a common feature in majority of solid tumors supports more aggressive disease, and acts as a strong driving force in inducing survival responses. In comparison to the non-transformed cells, tumor cells tend to overcome cell-cycle arrest and sustain proliferation to thrive in the hypoxic tumor milieu [27]. We have elucidated the role of MK2 in regulating the mRNA turnover by reporting that MK2 controls the stability of TNF-α, VEGF, p27 and MKP-1 transcripts in tumor microenvironment. MK2_KD_ destabilized TNF-α and VEGF transcripts while increase in t_1/2_ of p27 and MKP-1 transcripts established that in addition to changing the transcriptional landscape of mRNAs, MK2 is critically involved in regulation of HNSCC pathogenesis. To the best of our knowledge, this is the first study detailing the p38-mediated signalling leading to MK2 activation and its putative role in HNSCC progression.

Recently it has been shown that post-transcriptional control of TNF-α synthesis is mainly MK2-mediated via AREs in the 3′-UTRs of its mRNA [28]. Here, we report that TNF-α transcripts are destabilized in MK2_KD_ cells. Our findings are consistent with a past report which shows that MK2 deficiency down-regulates TNF-α production [29]. Investigations in the past have suggested the involvement of MK2 in VEGF-induced cell migration [30]. Many reports have proposed MK2-RBPs mediated stabilization and elevation of VEGF expression in hypoxic tumors [9]. On this line, our results demonstrated that MK2_KD_ facilitates post-transcriptional decay of VEGF mRNA that supports the above mentioned role of MK2 in the regulation of VEGF in tumors. Our hypothesis is further strengthened by reports that suggested impairment in the inflammatory response in MK2-deficient mice [31].

p27, a critical factor for controlling cellular proliferation, functions as a tumor suppressor with reduced expression associated with poor patient survival. Moreover, its loss has been implicated in tumorigenesis and is linked to a more severe phenotype [32]. MAPK pathway seems to be involved in the negative control of this inhibitor by augmenting its degradation, thereby presumably supporting unrestricted cell growth. Our findings agree well with this hypothesis, as we have reported that MK2_KD_ tends to stabilize p27 by increasing t_1/2_ of its transcripts. Various reports confirmed the fact that in aggressive tumors like HNSCC, low levels of p27 are due to its decreased stability, hence, further validating our findings [33]. Through its phosphatase action, MKP-1 regulates the magnitude and duration of MAPK signaling through negative-feedback regulation, and it has been well reported that the short-lived MKP-1 mRNA is rapidly induced by different stresses [34]. Our results postulated that MK2_KD_ tends to stabilize the MKP-1 transcripts, thus, confirming the hypothesis that mRNA stabilization of this negative regulator possibly inhibits the progression of HNSCC. Our findings are in agreement with the latest report showing that systemic MK2 deletion reduced tumor burden by immunomodulatory cancer therapies in mice [35].

Murine models are being extensively utilized to enhance our knowledge about the mechanisms of tumor pathogenesis [36]. Several studies have reported that subcutaneous xenograft models can predict the clinical activity of cytotoxic agents as potential anti-cancer drugs and serve as model systems to better understand tumor progression [37, 38]. Importance of mesenchymal MK2 inhibition in colorectal tumor development is already known [39]. Here, using a xenograft NOD/SCID mice model we confirm that the tumors that develop in vivo in MK2-KD scenario grow slower and are less aggressive pointing towards a clear role of MK2 in modulating HNSCC pathogenesis. Inhibition of the p38/MK2 pathway by blocking p38 failed, as none of the p38 inhibitors were found successful in the clinical trials due to the unwanted side effects [6]. For this reason, in recent years, MK2 was selected as a potential candidate for targeted therapies as an alternative to p38 in an attempt to abrogate the systemic side effects associated with p38 inhibitors. MK2 inhibition has been shown to have the advantage of lacking side effects possibly without any rebound effect [6].

Given the pivotal importance of the p38/MK2 pathway in inflammation, cell-cycle and metastasis, MK2 still remains a very promising target. Lately, the identification of drug-like MK2 inhibitors with appropriate pharmacokinetics and pharmacodynamics has been an appealing challenge for medicinal chemists. Our study has certain limitations which we would like to discuss too. First of all, due to the lack of patient data; staging, grading and survival curve analysis was not possible which could give a better picture of the role of MK2 in patient survival. Further, the progression of HNSCC also involves invasion and metastasis in combination with proliferation. Hence, future studies pertaining to the role of MK2 in these pathogenic pathways could be performed along with the demonstration of biological effects of MK2_KD_.

## Conclusion

Taken together, our findings demonstrate a clear link of MK2 in regulating HNSCC progression. This can contribute significantly to the understanding of inhibiting malignant development by controlling MK2 signaling, thereby, unveiling the importance of MK2 to functionally modulate HNSCC pathogenesis. Thus, MK2 can be taken forward as an alternative potential therapeutic target to the p38/MAPK mediated interventions in limiting HNSCC progression.

## Additional file

Additional file 1: This article contains additional files including supplemental experimental procedures, nine figures and eleven tables which can be accessed online. (PDF 2540 kb)

## Abbreviations

3′-UTR: 3′-untranslated region
ActD: Actinomycin D
ARE: Adenine/uridine-rich element (s)
ATCC: American Type Culture Collection
AUF1: AU-rich element binding factor-1
CEBPδ: CCAAT/enhancer-binding protein delta
CUGBP1: CUG triplet repeat RNA binding protein-1
DMEM: Dulbecco’s modified eagle’s medium
ECL: Enhanced chemiluminescence
EMEM: Eagle’s minimum essential medium
FBS: Fetal bovine serum
FFPE: Formalin-fixed and paraffin-embedded
GFP: Green fluorescent protein
H&E: Hematoxylin and eosin
HIF1-α: Hypoxia-inducible factor 1-alpha
HNSCC: Head and neck squamous cell carcinoma
HRP: Horseradish peroxidase
HuR: Human antigen R
IAEC: Institutional animal ethics committee
IEC: Institutional ethics committee
IHC: Immunohistochemistry
MAPKAPK2 or MK2: Mitogen-activated protein kinase-activated protein kinase 2
MK2_KD_: MK2-knockdown
MK2_WT_: Wild-type MK2
MKP-1: Mitogen-activated protein kinase phosphatase-1
NOD/SCID: Non-obese diabetic/ severe combined immunodeficiency
p27: Cyclin-dependent kinase inhibitor 1B
qRT-PCR: Quantitative real time-polymerase chain reaction
RBP: RNA-binding protein (s)
shRNA: Short hairpin RNA
SRB: Sulforhodamine B
TNF-α: Tumor necrosis factor-alpha
TTP: Tristetraprolin
VEGF: Vascular endothelial growth factor
WB: Western blotting
