# Supplementary material for: MAPKAPK2 plays a crucial role in the progression of head and neck squamous cell carcinoma by regulating transcript stability
Source: J Exp Clin Cancer Res. 2019 Apr 25;38:175. doi: 10.1186/s13046-019-1167-2 (PMC6482562; doi:10.1186/s13046-019-1167-2)
Supplement: Supplementary file 1 — This article contains additional files including supplemental experimental procedures, nine figures and eleven tables which can be accessed online. (PDF 2540 kb) [file 13046_2019_1167_MOESM1_ESM.pdf]

ADDITIONAL FILE 1

Immunohistochemistry

The levels of expression and activation status of specific proteins (listed in Additional file 1: Table S2) in procured clinical samples were analyzed using immunohistochemical (IHC) staining. Briefly, sections (5µm) were fixed on poly-L-lysine coated slides, followed by deparaffinization and rehydration. Antigen retrieval was performed using sodium citrate buffer (pH 6.0) followed by quenching of endogenous peroxidases using BLOXALL blocking solution (Vector Labs). Further, incubation of the sections with 2.5% normal horse serum blocked the exposed sites. Sections were reacted with appropriately diluted specific primary antibody overnight followed by incubation with HRP-conjugated secondary antibody for 1 hr. Rinsed sections were then incubated with 3,3'-diaminobenzidine (DAB) substrate, Mayer's hematoxylin was employed as a counterstain. Observations were carried out under bright field microscope. Staining intensity was semi-quantitatively graded by a pathologist in a blinded fashion.

Western blotting

For protein expression analysis, clinical tissue samples/cultured cells were lysed in protein lysis buffer containing RIPA buffer (150mM sodium chloride, 1.0% IGEPAL® CA-630, 0.5% sodium deoxycholate, 0.1% sodium dodecyl sulphate (SDS), 50mM Tris, pH 8.0, Sigma Aldrich), 10% protease inhibitor cocktail and 1% Triton X-100. Pre-chilled lysis buffer was added on to the cells followed by scraping and collection of the cells in micro-centrifuge tubes. Tissue samples were first dissected on ice, kept in micro-centrifuge tubes and then snap-frozen by submerging in liquid nitrogen. This was followed by the addition of pre-chilled lysis buffer and homogenization. The contents in micro-centrifuge tubes were

vortexed for about 1 hr (30 sec agitation followed by 5 min incubation at 4°C) followed by centrifugation at 12,000 rpm for 20 min at 4°C.

The lysate so obtained was quantified for total protein content using the Bradford assay. An equal amount of protein samples (50μg) were added into 2x Laemmli's-SDS sample buffer (65.8mM Tris-HCl, pH 6.8, 26.3% (w/v) glycerol, 2.1% SDS, 0.01% bromophenol blue and 355mM 2-mercaptoethanol) followed by boiling at 95°C for 5 min. The lysates were briefly centrifuged and resolved by SDS-polyacrylamide gel electrophoresis (12% acrylamide), and proteins were transferred from the gels to the membrane using Trans-Blot® Turbo™ RTA Mini Polyvinylidene fluoride (PVDF) Transfer Kit (Bio-Rad). The membrane was blocked by 5% non-fat dried milk for 1 hr at room temperature. Desired proteins on the membrane were probed with specific primary antibodies (appropriately diluted) and incubated overnight at 4°C (dilutions are mentioned in Additional file 1: Table S2). The membrane was washed six times (5 min each) with 1x Phosphate Buffered Saline with Tween 20 (PBST) and incubated with secondary anti-mouse or anti-rabbit IgG (H+L)-HRP antibody (Bio-Rad, 1:3000 dilution in 1x PBST) for 1 hr at room temperature. Expression of β tubulin was used as an internal loading control. Protein bands were detected and visualized using Clarity™ Western enhanced chemiluminescence (ECL) Substrate (Bio-Rad) with ECL imager (Azure).

Sulforhodamine B (SRB) assay

Sulforhodamine B colorimetric assay was performed for cytotoxicity analysis of Actinomycin D (ActD, Sigma Aldrich). CAL27 cells were seeded in 96-well plates ($2x10^4$ cells per well) and exposed to different concentrations of ActD (0.5, 1, 2.5, 5 and 10μM) for 24, 48 and 72 hrs. Post-treatment the cells were fixed with 50μl of 50% trichloroacetic acid (Merck) for 1 hr at 4°C. Further, the fixed cells were washed five times with water. After air drying, cells were

incubated in 100μl 0.4% SRB (Sigma Aldrich) for 30 min in the dark. Cells were then washed with 1% acetic acid five times to remove unbound dye. After air drying, 100μl of 10mM Tris base (pH 10.5) was added to dissolve the bound dye. Cytotoxicity was evaluated by optical density determination at 540nm using a microplate reader.

Transfection of CAL27 cells and shRNA knockdown experiments

CAL27 cells at about 60% confluence, cultured in specific growth medium were transfected with the aid of Attractene reagent (Qiagen) with psi-U6.1 vectors expressing different 19-mer MK2-specific shRNA constructs (Additional file 1: Figure S1 and Table S3). Further, a non-specific scrambled control shRNA in psi-U6 vector (Genecopoeia) was used, and the transfection was performed as per protocol recommended by the manufacturer (Qiagen). Briefly, transfection complexes incubated for 20 min at room temperature and containing a mixture of shRNA constructs (4μg) and Attractene transfection reagent were applied to the pre-seeded cells. Transfected cells were cultured for atleast 48 hrs before being passaged into puromycin selection medium (1μg/ml puromycin, Sigma Aldrich) to obtain stable transfectants.

RNA isolation

Total RNA was extracted from procured tissue samples and cell lines using RNeasy mini kit (Qiagen) following manufacturer's protocol. Briefly, RLT lysis buffer containing β-mercaptoethanol was used for cell lysis and the homogenate prepared was centrifuged at 10,000 rpm for 3 min. An equal volume of 70% ethanol was added to the supernatant, and the mixture was kept at room temperature for 5 min before loading to the RNeasy mini spin column. The mixture was centrifuged at 10,000 rpm for 30 sec, and the column was then washed with buffer RW1 followed by centrifugation. Further, two washings with RPE buffer were given to the column. Finally, RNA was eluted in nuclease-free water after dry spin.

Extracted RNA was quantified by spectrophotometric measurement using Nanodrop (Thermo Fisher Scientific).

Quantitative real time-PCR (qRT-PCR)

Extracted RNA was used for qRT-PCR analysis using Verso One-Step SYBR qRT-PCR kit, (Invitrogen) according to manufacturer recommended protocol. Primers used for all the selected human genes were custom synthesized and obtained from Integrated DNA Technologies (Supplementary Table S4) while TaqMan probes and primers from Applied Biosystems (Supplementary Table S5). The thermal cycling protocol employed is as follows: 50°C for 15 min (cDNA synthesis), 95°C for 15 min (enzyme activation), followed by 40 cycles of denaturing at 95°C for 15 sec, annealing at T_a for 30 sec and extension at 72°C for 30 sec and a standard melt curve in an automated sequence detection system (StepOnePlus Real-Time PCR System, Thermo Fisher Scientific). VeriQuest Probe One-Step qRT-PCR Kit (Affymetrix) was used for qRT-PCR analysis using TaqMan chemistry following manufacturer's protocol. qRT-PCR was carried out using the StepOne Plus Real-Time PCR System (Applied Biosystems) with the following protocol: 50°C for 15 min (reverse transcription), 95°C for 10 min (enzyme activation), followed by 40 cycles of denaturing at 95°C for 15 sec, annealing and extension at 60°C for 1 min. Relative gene expression was obtained after normalization with endogenous expression of human GAPDH and determination of the difference in threshold cycle (Ct) for cancer and normal tissues was carried out using the $2^{-\Delta\Delta Ct}$ method. All the qRT-PCR assays were performed atleast thrice.

Figure Legends:

Figure S1: Pictorial representation of shRNA construct. Vector information for human MK2-specific shRNA construct used for CAL27 transfection and subsequent knockdown of MK2 (Image source: GeneCopoeia)

Figure S2: Representative bright field micrographs of hematoxylin and eosin (H&E) stained sections of human clinical tissue samples confirming HNSCC. (A) Normal head and neck region tissue sections marked by the presence of tissue fragment lined by stratified squamous epithelium (indicated by arrows) without any dysplastic or anaplastic features. (B) HNSCC tissue sections containing squamous cells with prominent nucleoli and extensive proliferation (indicated by arrows) and invasion into the sub-epithelial region. The images have been captured at 100x (original magnification) and 200x (insets).

Figure S3: Relative gene expression levels of HNSCC pathogenesis-specific genes in clinical tissues resulting from qRT-PCR analysis using SYBR Green chemistry. Graphical representation of qRT-PCR results showing the relative gene expression of various genes involved in HNSCC pathogenesis as compared to control samples (using SYBR Green chemistry). Histograms represent the levels of: **(A)** Top 5 up-regulated and **(B)** Top 5 down-regulated genes. Relative gene expression was obtained after normalization with endogenous human GAPDH and determination of the difference in threshold cycle (Ct) between tumor and normal tissues was performed using the $2^{-\Delta\Delta Ct}$ method. All the qRT-PCR assays were performed in triplicate. The results are expressed as means±standard errors of the mean. ***, $p<0.001$ and ****, $p<0.0001$ represent the statistical significance compared with control.

Figure S4: Relative gene expression levels of HNSCC pathogenesis-specific genes in clinical tissues resulting from qRT-PCR analysis using TaqMan chemistry. Graphical representation of qRT-PCR results showing the relative gene expression of various genes

involved in HNSCC pathogenesis as compared to control samples (using TaqMan chemistry). Histograms represent the levels of: **(A)** Up-regulated and **(B)** Down-regulated genes. Relative gene expression was obtained after normalization with endogenous human GAPDH and determination of the difference in threshold cycle (Ct) between tumor and normal tissues was performed using the $2^{-\Delta\Delta Ct}$ method. All the qRT-PCR assays were performed atleast thrice. The results are expressed as means±standard errors of the mean. *, $p<0.05$ and ****, $p<0.0001$ represent the statistical significance compared with control.

Figure S5: Validation of shRNA-GFP construct transfection into CAL27 cells and MK2 knockdown. Confirmation of CAL27 transfection with specific shRNA-GFP constructs using visual analysis from images captured *via* **(A)** Immunofluorescence microscope (Carl Zeiss), **(B)** Imaging flow cytometer (Amnis, Merck) and **(C)** EVOS FL Auto 2 Imaging System (Thermo Fisher Scientific). The images have been captured at 220x (10x objective), 440x (20x objective) and 880x (40x objective). Scale bar denotes 275µm (10x), 125µm (20x) and 75µm (40x).

Figure S6: Ascertaining the role of MK2 in regulation of HNSCC pathogenesis. qRT-PCR analysis of the same gene set that showed significant up/down-regulation in presence of MK2 was performed in CAL27-$MK2_{KD}$ cells. The results are presented in histograms. (A) SYBR Green chemistry. (B) TaqMan chemistry. Relative gene expression was obtained after normalization with endogenous human GAPDH and determination of the difference in threshold cycle (Ct) between $MK2_{KD}$ and normal CAL27 cells was performed using the 2-ΔΔCt method. All the qRT-PCR assays were performed in triplicate. The results are expressed as means±standard errors of the mean. SC is Scrambled Control transfected; shRNA 2 is shRNA-GFP construct 2 transfected and shRNA-Mix=Co-transfected with

shRNA-GFP constructs 1, 2, 3, 4. ****, $p<0.0001$ represent the statistical significance compared with control.

Figure S7: Increased gene expression of HIF-1α validated generation of hypoxia. qRT-PCR analysis was performed in RNA samples extracted from hypoxia exposed CAL27 cells. Histogram shows the level of relative gene expression of HIF-1α in CAL27 cells exposed to hypoxic conditions (0.5% O_2) for 24 and 48 hrs as compared to non-hypoxic control cells. The relative fold change value of HIF-1α after 48 hrs of hypoxic exposure is ~6 which confirmed generation of hypoxia in cells. qRT-PCR assays were performed in triplicate. The results are expressed as means±standard errors of the mean. *, $p<0.05$ represent the statistical significance compared with control.

Figure S8: Western blot analysis showing the levels of expression of HIF-1α. Western blotting was performed to evaluate the expression of HIF-1α in extracts prepared from CAL27 cells exposed to hypoxic conditions (0.5% O_2) for 24 and 48 hrs as compared to normoxic controls. We observed a higher expression of HIF-1α in hypoxia exposed cells which affirmed the generation of hypoxia in these cells. β tubulin served as a loading control in this case.

Figure S9: Cytotoxicity analysis of Actinomycin D. SRB assay was performed to evaluate the cytotoxic potential of Actinomycin D (ActD) on CAL27 cells. Cells were exposed to different concentrations of ActD (0.5, 1, 2.5, 5 and 10μM) for 24, 48 and 72 hrs to evaluate its cytotoxicity. The histograms represent the percentage cytotoxicity values of different concentrations of ActD in treated CAL27 cells cultured for 24, 48 and 72 hrs post-treatment. The results are expressed as means±standard errors of the mean. Vinblastine served as a positive control in this assay.

Table S1: Tabular representation of clinical information of the individual patients.

a) Human clinical sample details

Reg. No.	Sex	Age	Region	Survival Status	Patient History	
6046-1	F	-	True vocal cord (TVC), Larynx	No info.	No info.	**28 Samples from Glottis region**
75336-9	F	65	Epiglottis	No info.	Cataract surgery, Septoplasty	
0819-8	F	85	TVC	Died	No info.	
60176-2	M	45	Epiglottis (vallecullae)	No info.	Smoker & Alcoholic	
64888-2	M	46	Vocal cord	No info.	No info.	
01190-0	M	50	Vocal cord	No info.	Smoker	
57570-7	M	53	Vocal cord		No info.	
2344-8	M	55	Epiglottis (vallecullae)	Died	Smoker & Alcoholic	
56329	M	57	Supraglottis/ TVC/trachea	No info.	Gout, Anti-Tubercular Treatment taken, Smoker & Alcoholic	
5862-6	M	58	Supraglottis	No info.	No info.	
65536-6	M	59	Glottis	No info.	No info.	
90528-9	M	60	Supraglottis	No info.	No info.	
62597-4	M	60	Supraglottis	No info.	No info.	
7357-1	M	62	Glottis	No info.	No info.	
67337-1	M	62	Glottis/vocal cord	Alive	Smoker & Alcoholic	
51186-0	M	65	Glottis/vocal cord	No info.	Anti-Tubercular Treatment taken	
-	M	65	Glottis	No info.	No info.	
7855-5	M	66	Epiglottis	No info.	No info.	
4769-7	M	67	Supraglottis/ hypopharynx	No info.	Smoker	
34566-8	M	68	Glottis	No info.	Cholecystectomy, Appendectomy, Smoker & Alcoholic	
54465-8	M	68	Supraglottis	No info.	No info.	
86862-1	M	68	Glottis	No info.	No info.	
2963-6	M	70	Supraglottis	Died	Smoker	
1571-4	M	70	Supraglottis/ pharynx	No info.	No info.	
64127-2	M	72	Glottis	No info.	No info.	
49592-1	M	72	Subglottis	No info.	No info.	
66511-7	M	72	Subglottis	Died	Smoker & Alcoholic	
61941-0	M	75	Supraglottis	No info.	No info.	

51271-7	F	8	Nasopharynx	No info.	No info.	**19-Pharynx region**
12297-0	F	39	Hypopharynx	No info.	Dental caries	
62101-4	F	60	Pharynx	No info.	No info.	
50922	F	64	Supraglottis/ oropharynx	No info.	No info.	
2393-4	F	70	Nasopharynx	No info.	No info.	
7920-0	F	70	Pharynx	No info.	No info.	
2658-8	M	40	Oropharynx	No info.	Psychological disorder, Smoker & Alcoholic	
2865-4	M	47	Pharynx	No info.	No info.	
65888-3	M	50	Oropharynx	No info.	No info.	
52684-7	M	50	Oropharynx	No info.	No info.	
52097-6	M	56	Hypopharynx	No info.	Chronic drug abuse, Smoker	
24411-1	M	56	Oropharynx	No info.	No info.	
60245-8	M	60	Hemilarynx	No info.	Smoker	
62747-1	M	66	Hypopharynx	No info.	No info.	
54382-1	M	68	Pharynx	Alive	Anti-Tubercular Treatment taken, Smoker & Alcoholic	
161592-1	M	69	Pharynx	No info.	Cataract surgery, Smoker	
41915-1	M	70	Oropharynx	No info.	No info.	
53837-6	M	70	Pharynx	No info.	No info.	
2066-7	M	71	Pharynx	No info.	Smoker	
-	F	28	Retro molar trigone (RMT)	No info.	No info.	**15-Tongue region**
19243-2	M	33	Papilloma tongue	No info.	No info.	
8388-2	M	46	Tongue	No info.	Smoker & Alcoholic	
49856-2	M	48	RMT	No info.	No info.	
31635	M	49	Base of tongue (BOT)	No info.	No info.	
20208-6	M	53	RMT	No info.	No info.	
37124-5	M	55	Tongue	No info.	No info.	
66173-6	M	56	RMT/tongue	No info.	No info.	
90370	M	60	RMT	No info.	No info.	
40618-3	M	61	Tongue	No info.	No info.	
61142-2	M	66	Tongue	No info.	No info.	
4485	M	70	BOT	No info.	No info.	
65543-8	M	70	Tongue	No info.	No info.	
64299-3	M	75	Tongue	No info.	No info.	
59571-1	M	76	Tongue	No info.	No info.	

4287-7	F	42	Nasal cavity	Died	Hypertension	**8 -Nasal region**
52892-8	F	45	Nasal cavity/ maxilla	Died	Smoker	
1841	F	48	Nasal cavity	Alive	Cholecystectomy	
28259-9	F	56	Nasal cavity	Alive	Uterus Fibroid	
28255	M	32	Nasal cavity	No info.	No info.	
56112-7	M	43	Nasal cavity	No info.	No info.	
52642-7	M	55	Nasal cavity/maxilla	Died	No info.	
54289-9	M	65	Nasal cavity	No info.	Smoker	
170411	M	50	Arytenoid/ larynx	No info.	Smoker	**5 -Larynx**
4838-8	M	57	Larynx (arytenoid)	No info.	No info.	
54737-5	M	57	Larynx	No info.	No info.	
3026-8	M	61	Larynx (arytenoid)	No info.	No info.	
612903-5	M	65	Larynx	No info.	No info.	
55459-8	F	30	Tonsil	No info.	No info.	**3**
41673-3	M	65	Tonsil	No info.	No info.	
3534-8	M	74	Tonsil	No info.	No info.	
637674	F	60	Maxilla	No info.	No info.	**2**
8601-2	M	60	Maxilla	No info.	No info.	
45236-4	M	19	Neck	No info.	No info.	**2**
53488-4	M	60	Neck	No info.	No info.	
60046-4	M	30	Lower lip	No info.	No info.	**2**
56394-1	M	45	Lip	No info.	No info.	
2220-7	M	79	Palate	No info.	No info.	**1**
53303-8	F	70	Gingiva	No info.	No info.	**1**
31301	F	45	Paranasal sinus	Alive	Hypertension	**1**
340938	M	43	Dermatofibrosarcoma	No info.	No info.	**1**
26094	F	26	Salivary gland	No info.	No info.	**7-Salivary Gland**
4606-0	F	29	Salivary gland	No info.	No info.	
1659-7	F	34	Salivary gland	No info.	No info.	
-	F	50	Salivary gland	Alive	No info.	
-	F	50	Salivary gland	Alive	Hypothyroidism	
-	M	-	Salivary gland	No info.	No info.	
74975-3	M	28	Salivary gland	No info.	No info.	
49983-9	M	70	Lymph node	No info.	No info.	**2**
55676-8	M	63	Lymph node	Died	Smoker & Alcoholic	

3697-6	F	28	Thyroid	No info.	No info.	**3**
8475-4	F	40	Thyroid	No info.	No info.	
1777	M	55	Thyroid	No info.	No info.	

b) FFPE blocks details

S. No.	FFPE Block type	No. of Blocks
1.	HNSCC	40
2.	Head and Neck Region Control	10

Table S2: Tabular representation of the various primary antibodies used for immunohistochemical staining and Western blot analysis along with the details of the dilutions used and other relevant information.

S.No.	ANTIBODY	DILUTION	RAISED IN	MAKE
**1.**	**p38/CRK (3G11C1)** (MA5-15891)	IHC-1:200 WB-1:1000	MOUSE MONOCLONAL	THERMO
**2.**	**Phospho-p38 (Thr180/Tyr182)** (MA5-15218)	IHC-1:200 WB-1:500	MOUSE MONOCLONAL	THERMO
**3.**	**MAPKAPK-2 (D1E11)** (12155S)	IHC-1:50 WB-1:1000	RABBIT MONOCLONAL	CST
**4.**	**Phospho-MAPKAPK-2 (Thr334)** (3007S)	IHC-1:50 WB-1:500	RABBIT MONOCLONAL	CST
**5.**	**C/EBP-δ Antibody (C-6)** (sc-365546)	IHC-1:50 WB-1:1000	MOUSE MONOCLONAL	SANTA CRUZ
**6.**	**Phospho-C/EBP-δ (Thr171)** (GTX116073)	IHC-1:50 WB-1:500	RABBIT POLYCLONAL	GENETEX
**7.**	**AUF1/hnRNPD (D6O4F)** (12382S)	IHC-1:50 WB-1:1000	RABBIT MONOCLONAL	CST
**8.**	**Phosho-AUF1/hnRNPD (Ser83)** (PA5-37596)	IHC-1:50 WB-1:500	RABBIT POLYCLONAL	THERMO
**9.**	**HuR (ELAVL1)** (MA5-18111)	IHC-1:100 WB-1:1000	MOUSE MONOCLONAL	THERMO
**10.**	**Phospho-HuR (Ser 221)** (ABE265)	IHC-1:50 WB-1:500	RABBIT POLYCLONAL	MERCK
**11.**	**CUGBP1** (MA1-16675)	IHC-1:100 WB-1:1000	MOUSE MONOCLONAL	THERMO
**12.**	**Tristetraprolin (TTP)** (AB124024)	IHC-1:50 WB-1:1000	MOUSE MONOCLONAL	ABCAM
**13.**	**HIF-1α** (MA116518)	IHC-1:100 WB-1:500	MOUSE MONOCLONAL	THERMO
**14.**	**β tubulin** (sc-58882)	WB-1:500	MOUSE MONOCLONAL	SANTA CRUZ

Table S3: Details of the human MK2-specific shRNA constructs used for transfection and subsequent knockdown of MK2 showing the MK2 binding location as well as the target sequence.

S.No.	shRNA	Clone Name	Location	Target Sequence
1.	shRNA 1	HSH022425-1-CU6(OS218617)	772	agcatccgaaatcatgaag
2.	shRNA 2	HSH022425-2-CU6(OS218618)	805	catccagtatctgcattca
3.	shRNA 3	HSH022425-3-CU6(OS218619)	1234	caccgagtttatgaaccac
4.	shRNA 4	HSH022425-4-CU6(OS218620)	1486	catctggtatcatcttctc
5.	Scrambled Control (SC)	CSHCTR001-1-CU6(OSNEG20)	-	gcttcgcgccgtagtctta

Table S4: Sequence details of the various human-specific primes used for qRT-PCR analysis (SYBR Green chemistry) in this study. Table also illustrates the primer length as well as the melting and annealing temperature of the primer sets used.

S. No.	Gene Name	Primer Sequence	Length	T_m (°C)	T_a (°C)
1.	GAPDH-F	CCATCTTCCAGGAGCGAGAT	20	56.4°C	55°C
	GAPDH-R	GCCTTCTCCATGGTGGTGAA	20	57.4°C	55°C
2.	COX-2-F	GCTCAGCCATACAGCAAATCC	21	56.5°C	55°C
	COX-2-R	CCGGGTACAATCGCACTTATA	21	54.4°C	55°C
3.	VEGF-F	CTACCTCCACCATGCCAAGT	20	56.9°C	55°C
	VEGF-R	GCGCTGATAGACATCCATGA	20	54.6°C	55°C
4.	GMCSF-F	GCAGCCTCACCAAGCTCAAG	20	58.9°C	55°C
	GMCSF-R	GTCTGGGTTGCACAGGAAGTT	21	57.7°C	55°C
5.	TNFα-F	CCCCAGGGACCTCTCTCTAA	20	57.5°C	55°C
	TNFα-R	TTGCTACAACATGGGCTACAG	21	55.1°C	55°C
6.	c-FOS-F	CAGACCGAGATTGCCAACCT	20	57.4°C	55°C
	C-FOS-R	AGGTCATCAGGGATCTTGCA	20	55.9°C	55°C
7.	C-MYC-F	CCAGAGGAGGAACAAGAAGATG	22	54.9°C	55°C
	C-MYC-R	CAGCAGAAGGTGATCCAGACT	21	56.5°C	55°C
8.	IL-1β-F	TGAAGCTGATGGCCCTAAACA	21	56.6°C	55°C
	IL-1β-R	GTAGTGGTGGTCGGAGATTCG	21	57.1°C	55°C
9.	IL-8-F	CCTTTCCACCCCAAATTTATC	21	51.6°C	55°C
	IL-8-R	GCTCTCTTCCATCAGAAAGCTT	22	54.9°C	55°C
10.	IL-6-F	TACCCCCAGGAGAAGATTCCA	21	57.2°C	55°C
	IL-6-R	CCGTCGAGGATGTACCGAATT	21	56.7°C	55°C
11.	MKP-1-F	CTCCACCACCACCGTGTTC	19	58.4°C	55°C
	MKP-1-R	GCTGGGAGAGGTCGTAATGG	20	57.4°C	55°C
12.	CYCLIN-A-F	ACAGCCTGCGTTCACCATTC	20	58.3°C	55°C
	CYCLIN-A-R	AAAAGCCAGGGCATCTTCAC	20	55.9°C	55°C
13.	CYCLIN-B1-F	CAGAAGATGGAGCTGATCCAA	21	54.4°C	55°C
	CYCLIN-B1-R	GTCTGACTGCTTGCTCTTCCT	21	56.9°C	55°C
14.	p27-F	AGCACTGCAGAGACATGGAA	20	56.5°C	55°C
	p27-R	TCCACCTCTTGCCACTCGTA	20	58.0°C	55°C
15.	HIF1α-F	ACACACAGCGAAGCTTTTTTC	21	54.4°C	55°C
	HIF1α-R	GTGCAGTGCAATACCTTCCAT	21	55.7°C	55°C
16.	CYCLIN-D1-F	CGCTGGCCATGAACTACCT	19	57.5°C	53°C
	CYCLIN-D1-R	TTAGAGGCCACGAACATGCA	20	56.8°C	53°C
17.	CATENIN-β1-F	GCTGGGACCTTGCATAACCTT	21	57.7°C	53°C
	CATENIN-β1-R	CTGGTGAACCAAGCATTTTCA	21	53.8°C	53°C
18.	TGFβ-F	GGGAACGCTTCGACAATGAG	20	56.2°C	53°C
	TGFβ-R	GTACGGCTGTCGAGCAGGAA	20	59.4°C	53°C
19.	CYCLIN-A2-F	CAGTAAACAGCCTGCGTTCA	20	55.6°C	53°C
	CYCLIN-A2-R	CAGGGCATCTTCACGCTCTA	20	56.8°C	53°C
20.	C-JUN-F	GCTGGAGCGCCTGATAATC	19	56.0°C	53°C
	C-JUN-R	CTCCTGCTCATCTGTCACGTT	21	56.9°C	53°C
21.	Nucleolar PhosphoproteinB23-F	GGATGAGTTGCACATTGTTGA	21	53.6°C	53°C
	Nucleolar PhosphoproteinB23-R	AACCGTTGGCTGTACAGACAT	21	56.7°C	53°C
22.	CYCLIN-E1-F	CCGAGCAAAGAAAGCCATGT	20	56.1°C	54°C
	CYCLIN-E1-R	CCCGCTGCTCTGCTTCTTAC	20	58.3°C	54°C
23.	p53-F	CCCCTCTGAGTCAGGAAACAT	21	56.4°C	54°C
	p53-R	AGCATCAAATCATCCATTGCT	21	52.8°C	54°C

Table S5: Details of the TaqMan assays used for qRT-PCR analysis (using TaqMan chemistry) in this study.

Assay ID (Applied Biosystems)	Catalogue No.	Gene Symbol
Hs00900055_m1	4331182	VEGF
Hs01555410_m1	4331182	IL-1β
Hs01034249_m1	4331182	p53
Hs00355045_m1	4331182	Catenin β1
Hs00153277_m1	4331182	p27
Hs00174128_m1	4331182	TNF-α
Hs02786624_g1	4331182	GAPDH
Hs00996788_m1	4331182	Cyclin A2
Hs04194186_s1	4331182	c-Fos
Hs00610256_g1	4331182	MKP-1
Hs02339479_g1	4331182	Nu. Ph.

Table S6: The table represents the grouping of animals for the xenograft experiment. The NOD/SCID male mice were randomly divided into following 5 groups (based on the injection/grafting), each group containing 8 mice

Group Name	Group Description	No. of Mice
Group I (HEPM-X)	HEPM Xenograft (Normal Head and Neck Cells, Non-transfected)	8
Group II ($MK2_{WT}$-X)	CAL27-$MK2_{WT}$ Xenograft (Non-Transfected)	8
Group III ($MK2_{KD}$-X)	CAL27-$MK2_{KD}$ Xenograft (Transfected)	8
Group IV (NTC)	Normal Control (No treatment)	8
Group V (VC)	Vehicle Control (PBS)	8

Table S7: Tabular representation of the results obtained after qRT-PCR analysis (using SYBR Green chemistry) showing relative fold change of expression of selected genes in tumor samples as compared to normal controls. Up-regulated genes are highlighted in green while down-regulated genes in red. GAPDH was used as an endogenous control in this study.

S. No.	Genes	Average fold change	Status
	GAPDH	1	Control
1.	COX-2	~2	Up-regulation
2.	VEGF	~7	Up-regulation
3.	GM-CSF	~5	Up-regulation
4.	TNF-α	~5	Up-regulation
5.	IL-1β	~10	Up-regulation
6.	IL-8	~3.5	Up-regulation
7.	IL-6	~7	Up-regulation
8.	Cyclin A	~40	Up-regulation
9.	Cyclin B1	~12	Up-regulation
10.	HIF-1α	~3	Up-regulation
11.	TGF-β	~9	Up-regulation
12.	Cyclin A2	~20	Up-regulation
13.	c-Jun	~5	Up-regulation
14.	Nucleolar Phosphoprotein B23 (Nu. Ph.)	~7.5	Up-regulation
15.	Cyclin E1	~1.5	Up-regulation
16.	c-Fos	~0.4	Down-regulation
17.	MKP-1	~0.2	Down-regulation
18.	p27	~0.5	Down-regulation
19.	Catenin β1	~0.5	Down-regulation
20.	p53	~0.4	Down-regulation
21.	c-Myc	~0.7	Down-regulation
22.	Cyclin D1	~0.7	Down-regulation

Table S8: Tabular representation of the results obtained after qRT-PCR analysis (using TaqMan chemistry) showing relative fold change of expression of selected genes in tumor samples as compared to normal controls. Up-regulated genes are highlighted in green while down-regulated genes in red. GAPDH was used as an endogenous control in this study.

S. No.	Genes	Average fold change	Status
	GAPDH	1	Control
1.	VEGF	~6	Up-regulation
2.	IL-1β	~7	Up-regulation
3.	Cyclin A2	~22	Up-regulation
4.	TNF-α	~3.5	Up-regulation
5.	Nu. Ph.	~2	Up-regulation
6.	c-Fos	~0.1	Down-regulation
7.	MKP-1	~0.2	Down-regulation
8.	p27	~0.25	Down-regulation
9.	Catenin β1	~0.5	Down-regulation
10.	p53	~0.4	Down-regulation

Table S9: Tabular representation of the results obtained after qRT-PCR analysis (using both SYBR Green and TaqMan chemistry) showing relative fold change of expression of selected genes in CAL27-MK2$_{KD}$ cells as compared to non-transfected controls. Up-regulated genes are highlighted in green while down-regulated genes in red. GAPDH was used as an endogenous control in this study.

GENES	SYBR		TaqMan	
	shRNA 2	shRNA Mix	shRNA 2	shRNA Mix
VEGF	1.35	1.82	1.19	1.41
IL-1β	0.37	0.68	0.45	0.53
Cyclin A2	0.29	0.30	0.19	0.16
TNF-α	0.68	2.75	0.73	1.45
Nu. Ph.	0.47	0.51	0.32	0.21
c-Fos	2.94	10.9	1.33	7.16
MKP-1	1.48	3.62	1.55	6.76
p27	0.86	0.60	0.64	0.41
Catenin β1	0.93	1.46	0.71	0.70
p53	0.80	0.58	0.54	0.45

Table S10: Tabular representation of the gross tumor weight in CAL27-MK2$_{WT}$ and CAL27-MK2$_{KD}$ grafted mice groups. There were two cages per group (with 4 mice/cage). The tumors were resected 7 weeks post-grafting and then weighed.

Group			Tumor Gross Weight
**CAL27-MK2$_{WT}$**	**Cage 1**	**Mice 1**	200 mg
		**Mice 2**	200 mg
		**Mice 3**	140 mg
		**Mice 4**	2300 mg
	**Cage 2**	**Mice 1**	620 mg
		**Mice 2**	130 mg
		**Mice 3**	200 mg
		**Mice 4**	1080 mg
**CAL27-MK2$_{KD}$**	**Cage 1**	**Mice 1**	160 mg
		**Mice 2**	350 mg
		**Mice 3**	360 mg
		**Mice 4**	210 mg
	**Cage 2**	**Mice 1**	430 mg
		**Mice 2**	170 mg
		**Mice 3**	200 mg
		**Mice 4**	150 mg

Table S11: The different mice groups were assessed for their haematological parameters at the day of tumor resection. The table below is a representation of the results of few important haematological parameters. One-way ANOVA was used for calculation of significance amongst the groups. Columns not connected to the same alphabet are significant against each other (p<0.05).

	GROUP I Normal Control (No Treatment)	GROUP II Vehicle Control (PBS)	GROUP III HEPM Xenograft (Normal Head and Neck Cells)	GROUP IV CAL27-$MK2_{WT}$ Xenograft (Non-Transfected)	GROUP V CAL27-$MK2_{KD}$ Xenograft (Transfected)
**WBCs/µl**	2810 ± 520[b]	2910 ± 90[ab]	4270 ± 1320[a]	4260 ± 1420[a]	2890 ± 840[b]
**RBCs/µl**	8890000 ± 640000[b]	8550000 ± 180000[ab]	8380000 ± 630000[ab]	7870000 ± 870000[a]	9250000 ± 550000[b]
**Hemoglobin (g/dL)**	12.51 ± 0.92[ab]	12.40 ± 0.28[ab]	12.11 ± 0.85[a]	11.30 ± 1.21[a]	13.39 ± 0.78[b]
**Platelets/µl**	1473630 ± 408820[ab]	1721000 ± 186680[b]	1691290 ± 373110[b]	1749750 ± 283270[b]	1125000 ± 477220[a]
**Neutrophils (%)**	53.70 ± 4.63	60.55 ± 5.44	50.42 ± 9.30	51.55 ± 7.74	52.04 ± 10.14
**Lymphocytes (%)**	29.70 ± 5.56[ab]	22.10 ± 6.08[b]	31.62 ± 7.93[a]	35.56 ± 5.16[a]	31.64 ± 9.25[a]
**Monocytes (%)**	13.38 ± 3.14	13.25 ± 0.21	14.31 ± 2.47	10.56 ± 3.94	12.70 ± 3.39
**Eosinophils (%)**	3.06 ± 0.99[ab]	4.10 ± 0.85[b]	3.37 ± 0.82[ab]	2.33 ± 1.38[a]	3.63 ± 1.42[ab]

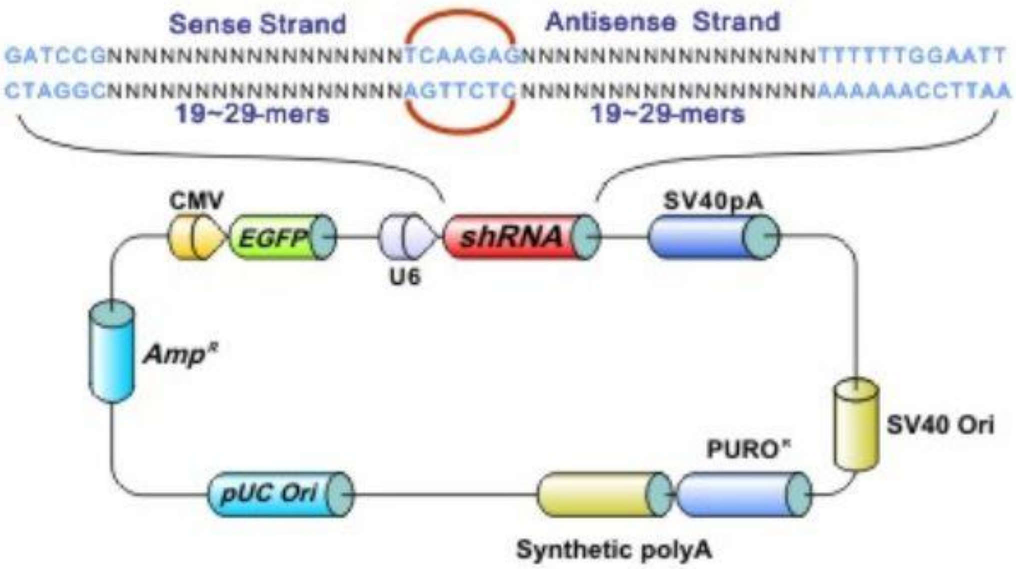
Sense Strand
Antisense Strand
GATCCGNNNNNNNNNNNNNNNNNNTCAAGAGNNNNNNNNNNNNNNNNNNTTTTTTGGAATT
CTAGGCNNNNNNNNNNNNNNNNNNAGTTCTCNNNNNNNNNNNNNNNNNNAAAAAACCTTAA
19~29-mers
19~29-mers
CMV
EGFP
U6
shRNA
SV40pA
AmpR
SV40 Ori
PURO
pUC Ori
Synthetic polyA

Figure S1

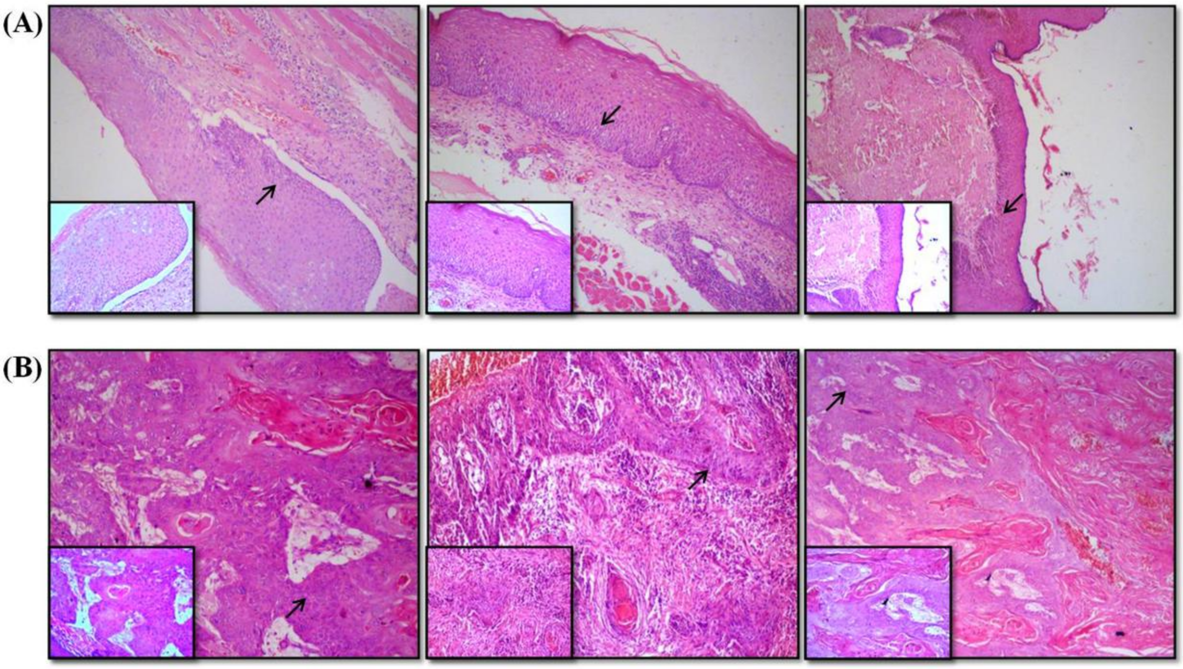

Figure S2

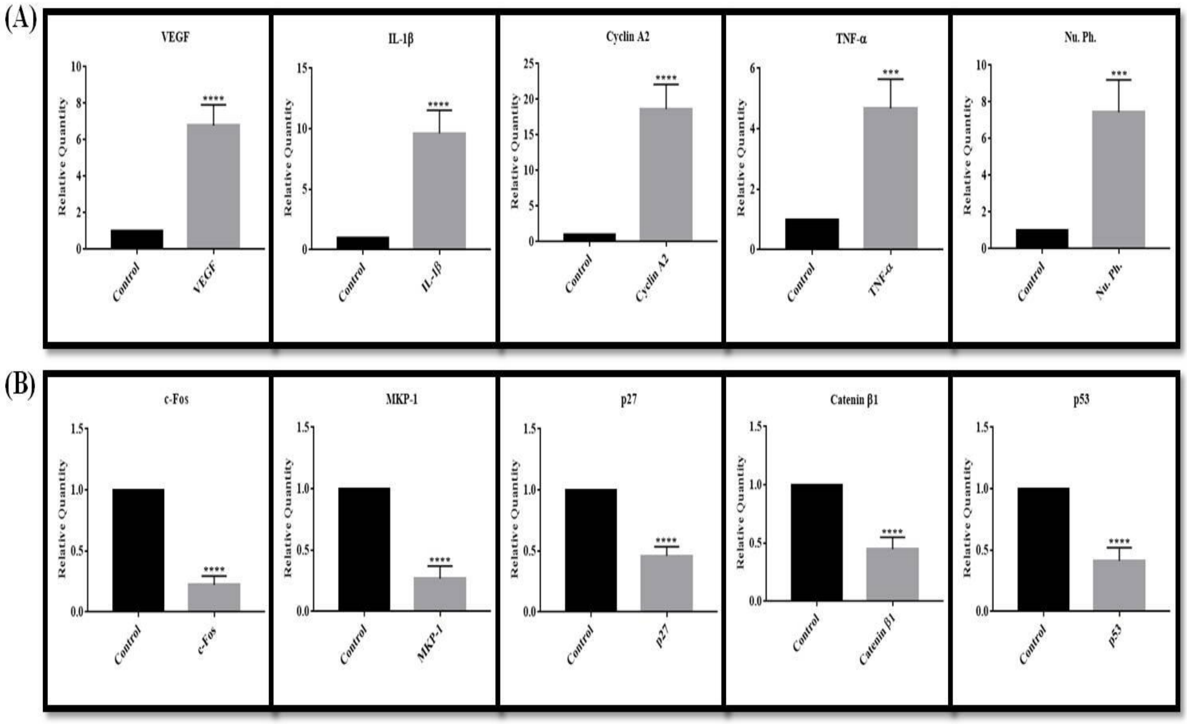
(A)
VEGF
IL-1β
Cyclin A2
TNF-α
Nu. Ph.
Relative Quantity
Control
****
***
(B)
c-Fos
MKP-1
p27
Catenin β1
p53
Relative Quantity
Control
****

Figure S3

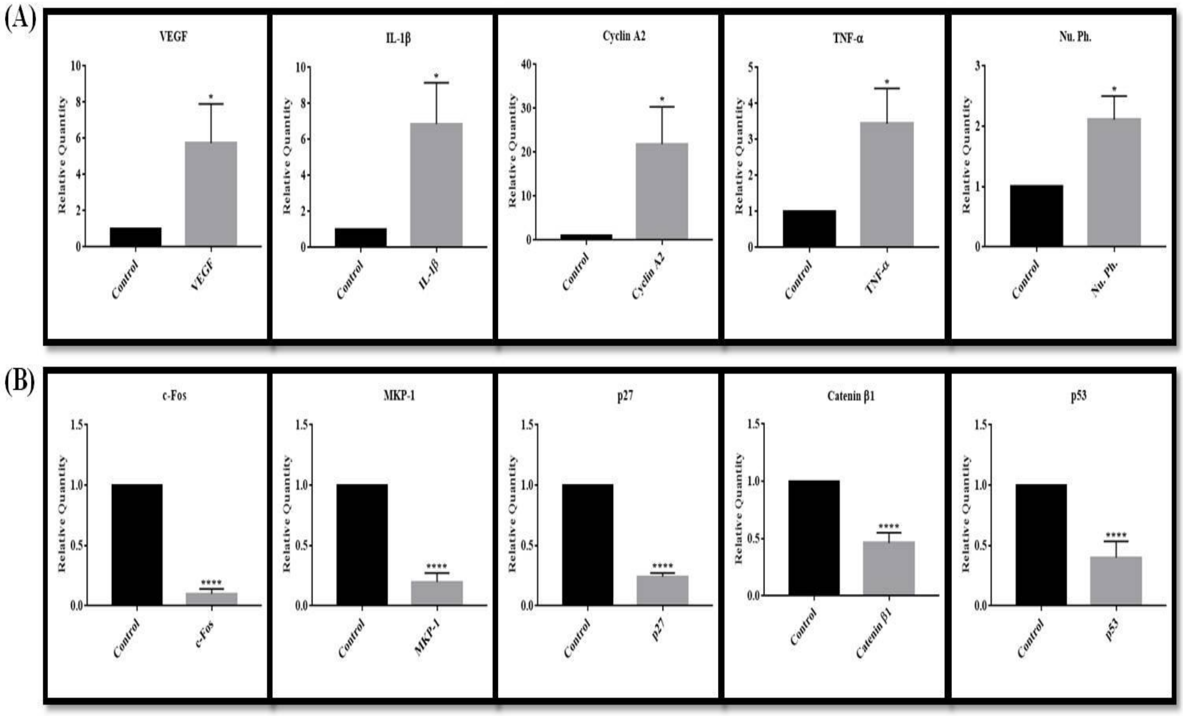
(A)
VEGF
IL-1β
Cyclin A2
TNF-α
Nu. Ph.
Relative Quantity
Control
(B)
c-Fos
MKP-1
p27
Catenin β1
p53
****

Figure S4

(A)

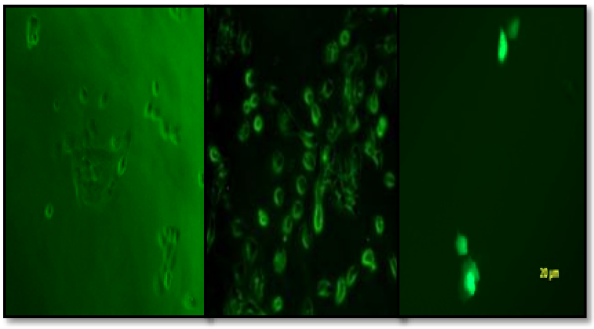

Non-transfected Transfected (10x) Transfected (20x)

(B)

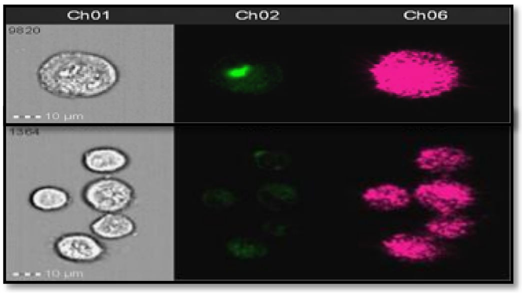

Brightfield FITC channel Side scatter

(C)

10x

20x

40x

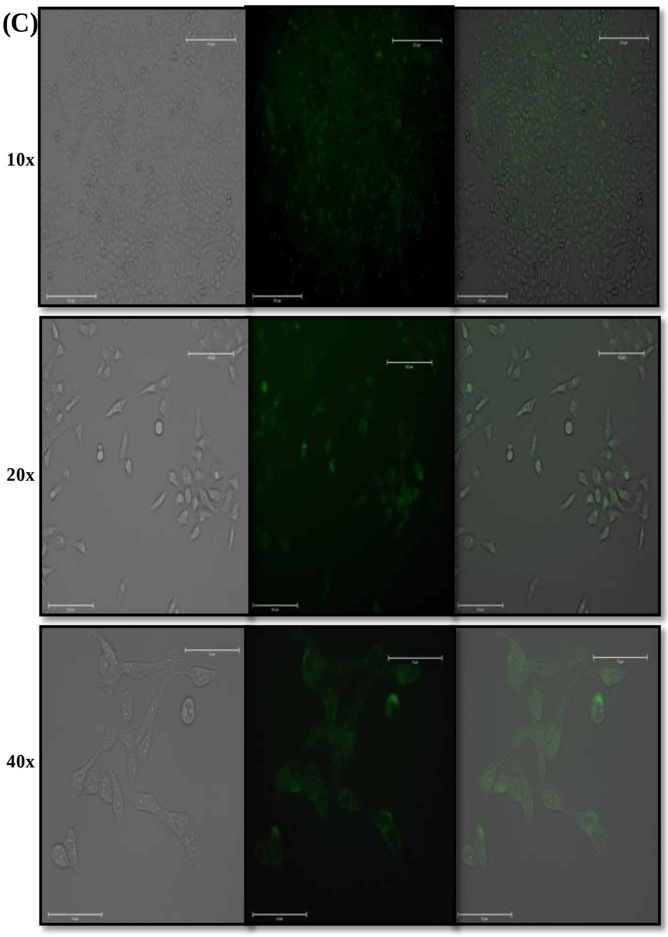

Brightfield FITC channel Merged

Figure S5

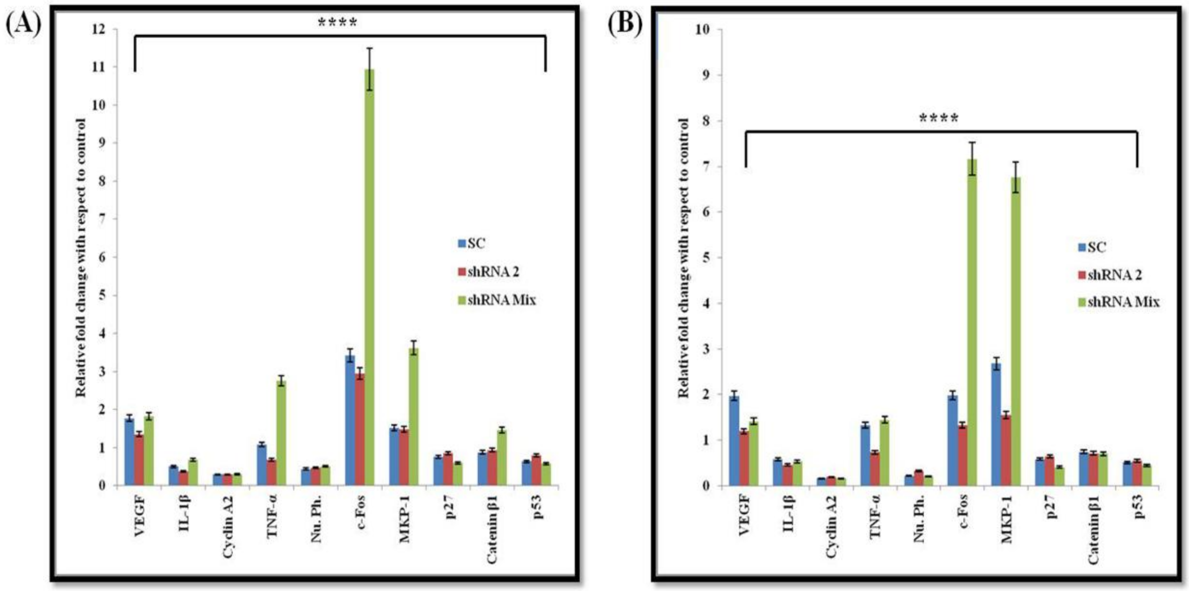
(A)
(B)
Relative fold change with respect to control
****
SC
shRNA 2
shRNA Mix
VEGF
IL-1β
Cyclin A2
TNF-α
Nu. Ph.
c-Fos
MKP-1
p27
Catenin β1
p53

Figure S6

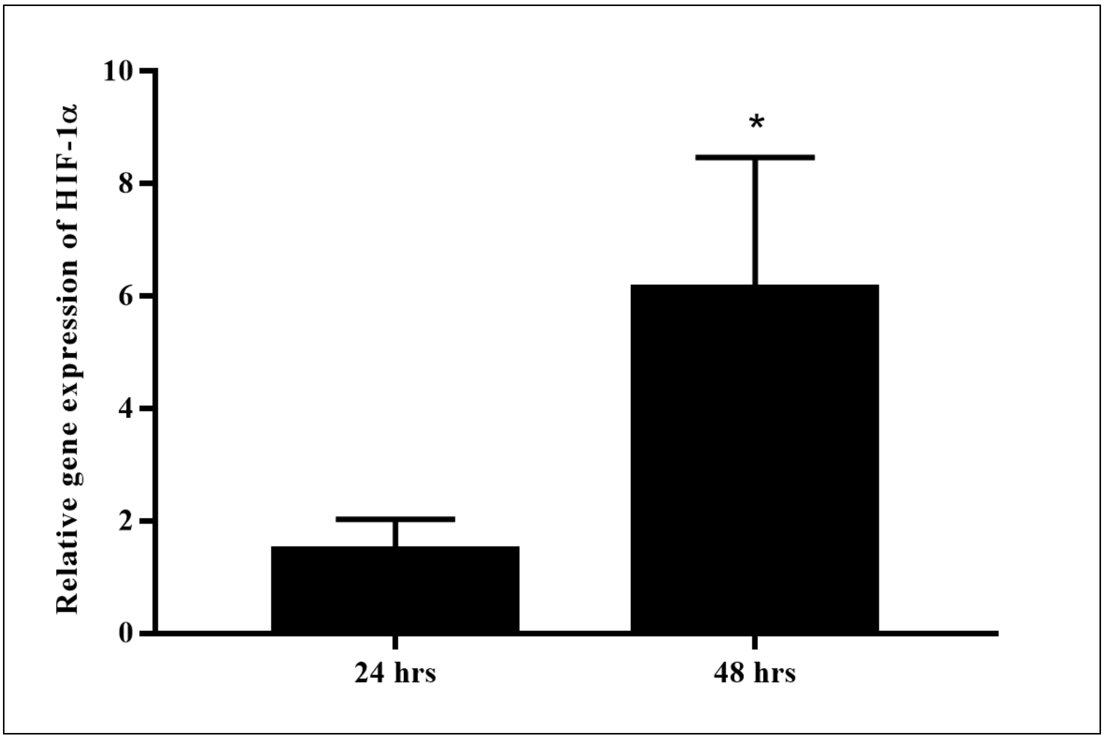

Figure S7

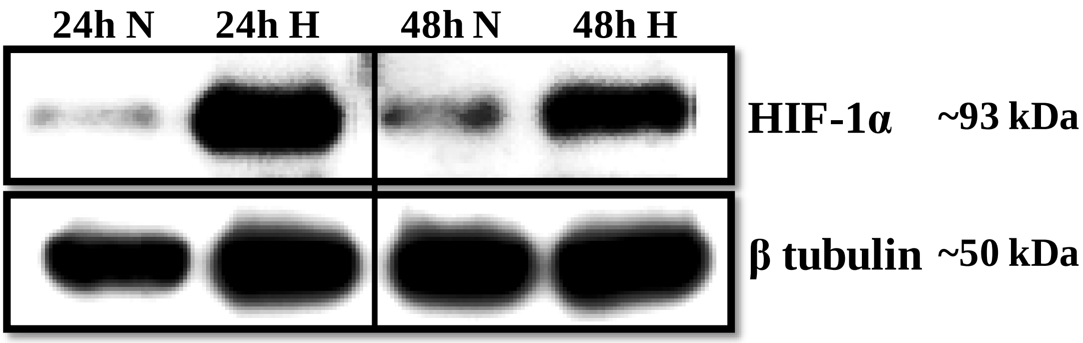

Figure S8

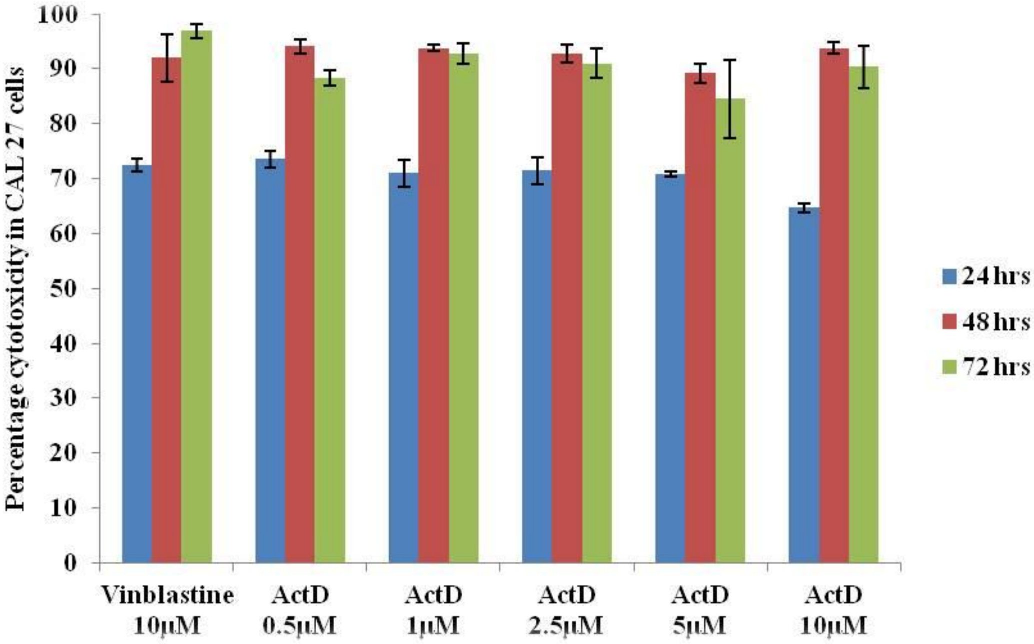
Percentage cytotoxicity in CAL 27 cells
100
90
80
70
60
50
40
30
20
10
0
Vinblastine 10µM
ActD 0.5µM
ActD 1µM
ActD 2.5µM
ActD 5µM
ActD 10µM
24 hrs
48 hrs
72 hrs

Figure S9

Enlarged images of Fig. 1 in main manuscript:

Panel A:

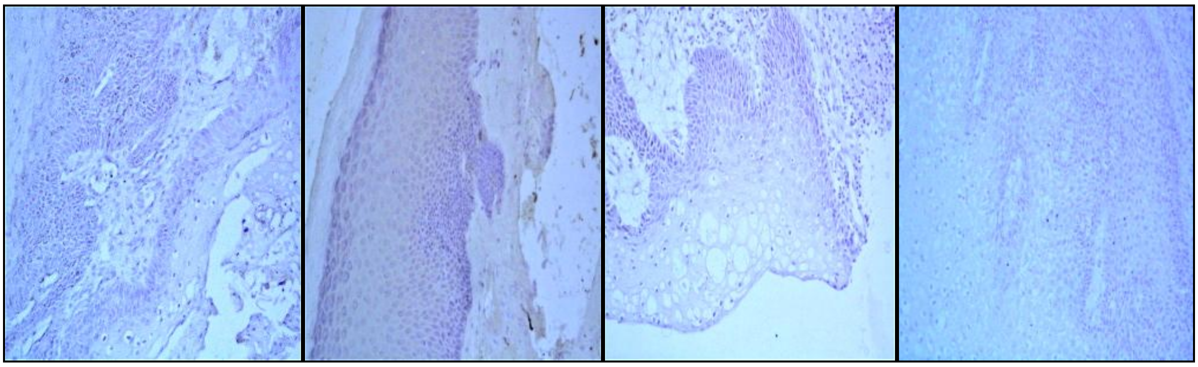

Panel B:

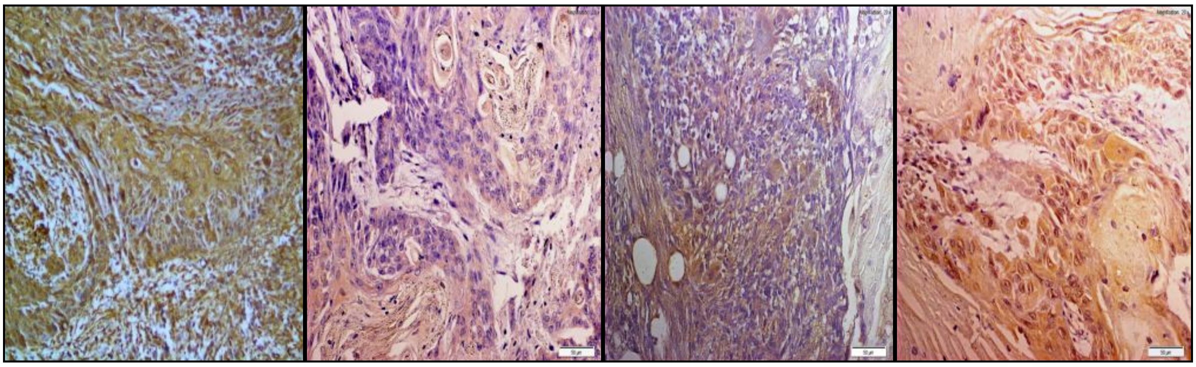

Panel C:

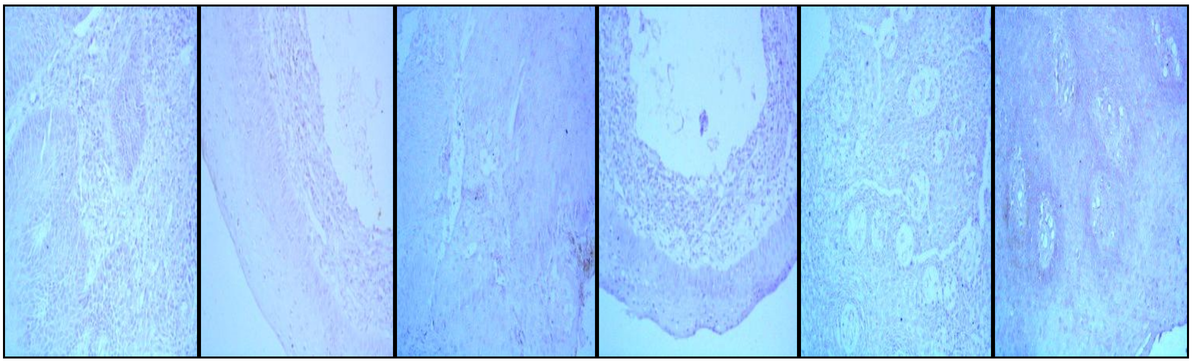

Panel D:

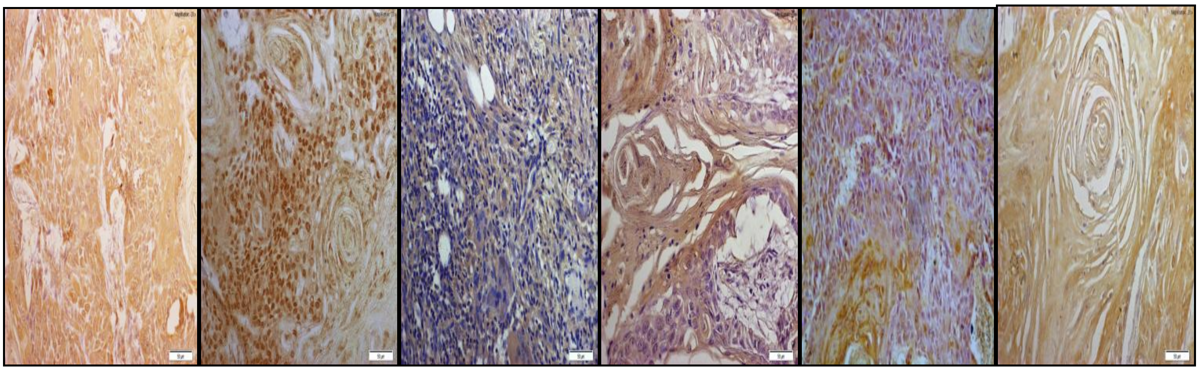

Panel E:

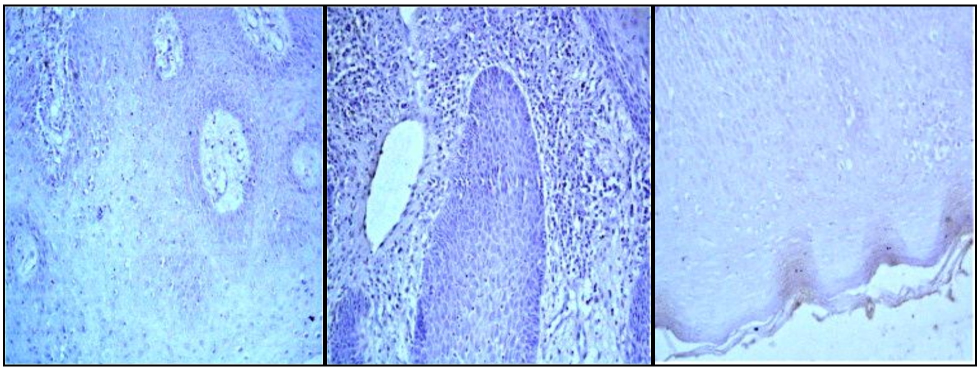

Panel F:

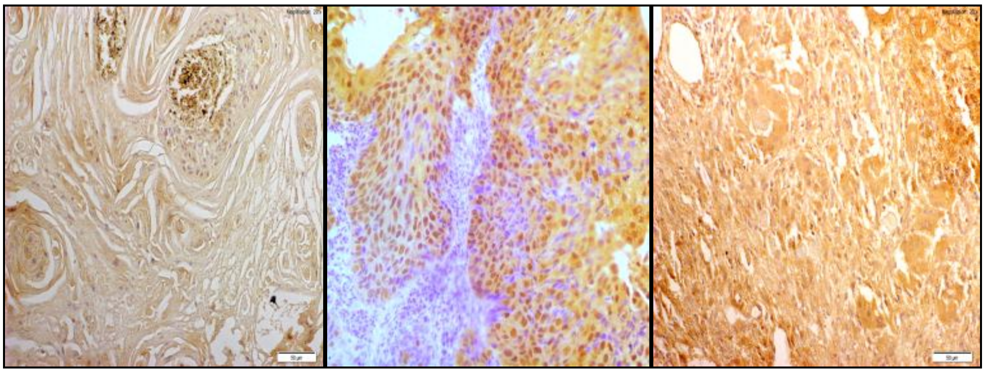

Figure 1: p38, MK2, RBPs and HIF-1α are overexpressed and activated in HNSCC. Representative IHC staining images of clinical tissue samples determining the expression and activation status of p38, p-p38, mitogen-activated protein kinase-activated protein kinase-2 (MK2), p-MK2, CCAAT/enhancer-binding protein delta (CEBPδ), p-CEBPδ, AU-rich element binding factor-1 (AUF1), p-AUF1, human antigen R (HuR), p-HuR, CUG triplet repeat RNA binding protein-1 (CUGBP1), tristetraprolin (TTP), and hypoxia-inducible factor-1 alpha (HIF-1α) in: **(A, C, E)** Normal tissue sections of head and neck region showing consistent negative staining in normal stratified squamous epithelium and, **(B, D, F)** HNSCC tissue sections showing consistent positive staining. The sections were subjected to IHC staining using specific primary antibodies followed by appropriate secondary antibody as described in Materials and Methods. Levels of expression of the above mentioned proteins were found to be relatively high in tumors (brown colour) as compared to normal controls. The images have been captured at 200x and the scale bar denotes 50μm.

Enlarged images of Fig. 2A in main manuscript:

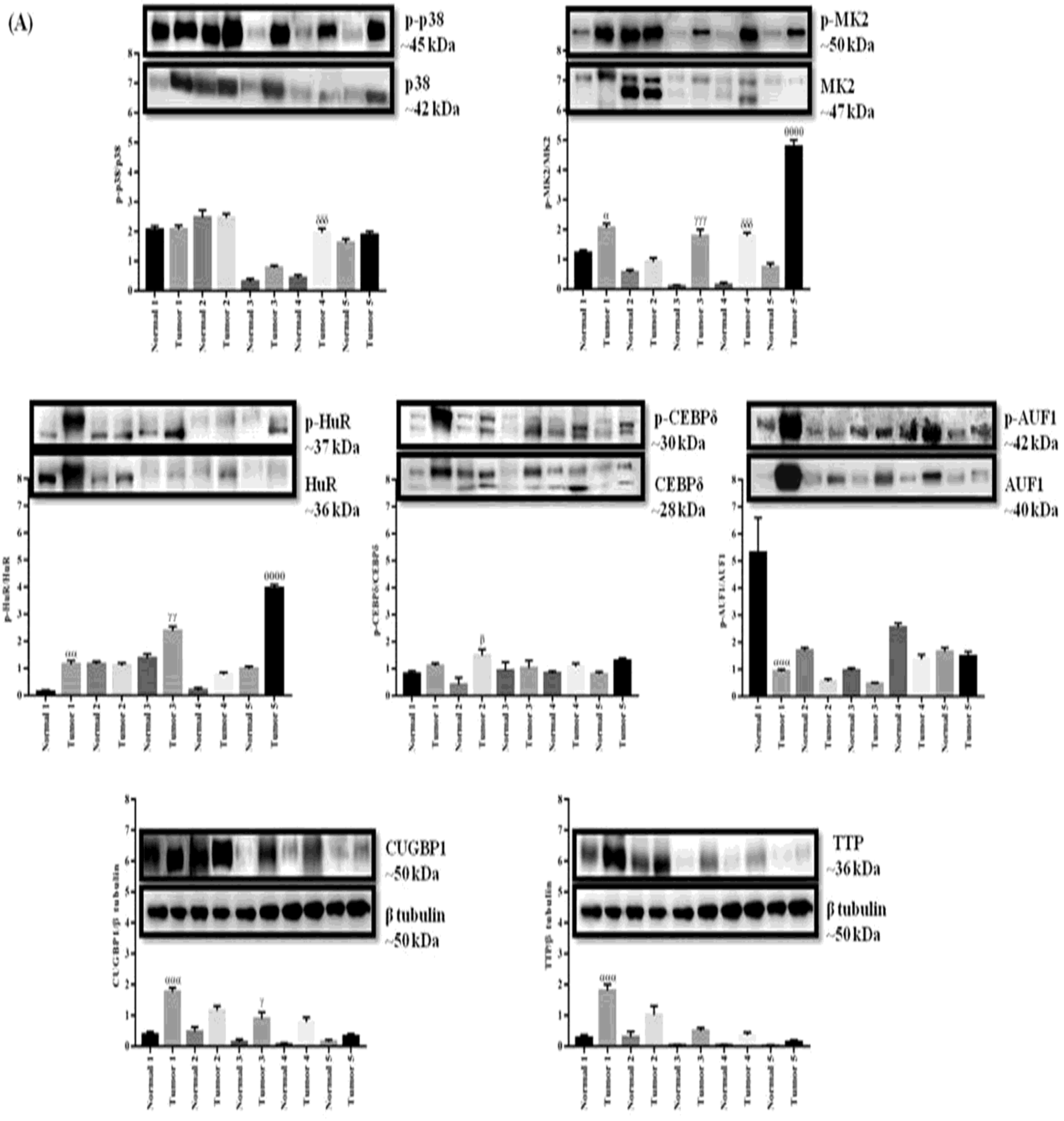

Enlarged images of Fig. 2B in main manuscript:

(B)

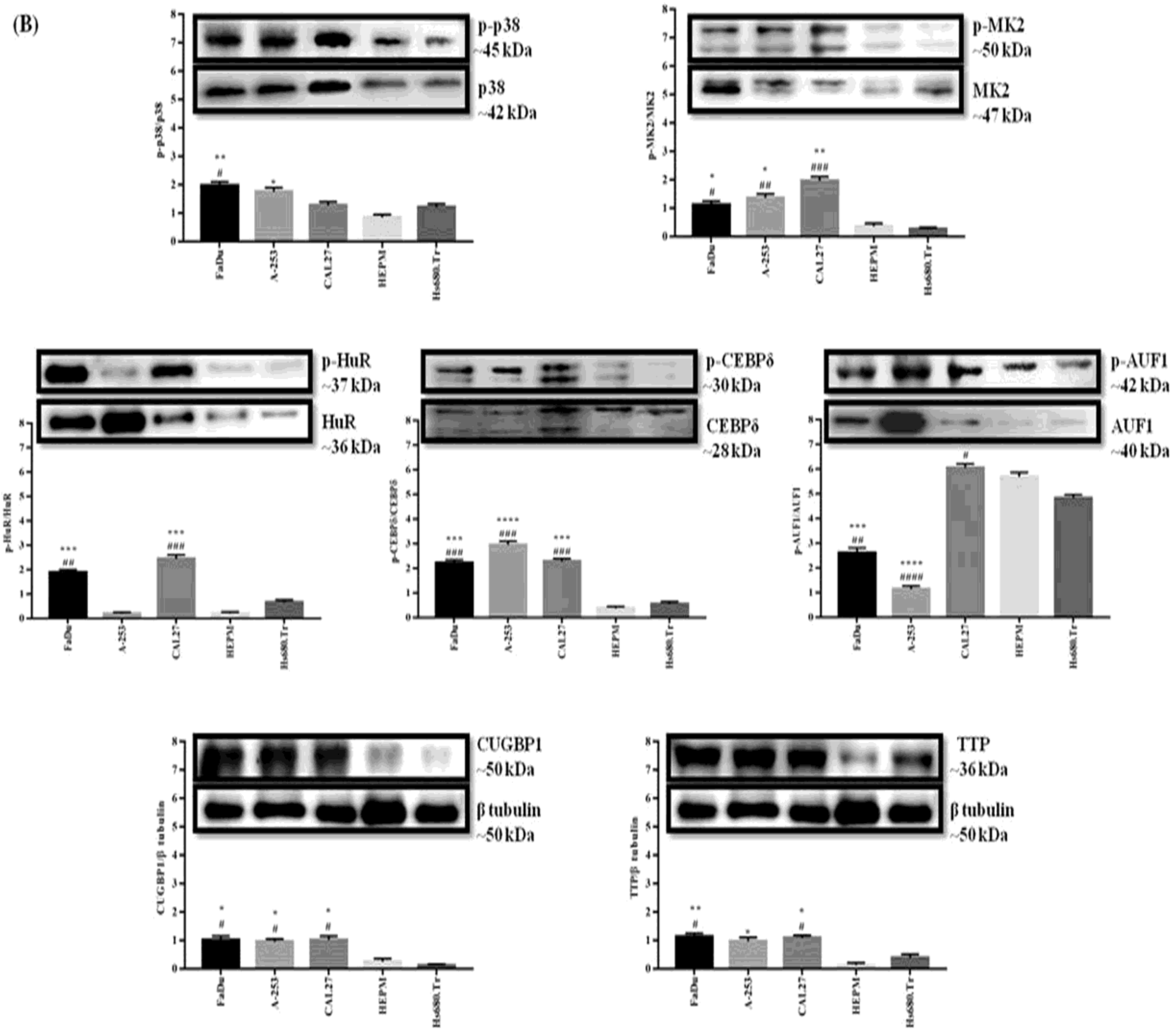

Figure 2: Western blot analysis confirmed higher levels of expression of specific proteins. Western blotting was performed to evaluate the levels of expression and activation status of p38, p-p38, MK2, p-MK2, HuR, p-HuR, CEBPδ, p-CEBPδ, AUF1, p-AUF1, CUGBP1 and TTP proteins in extracts prepared from: **(A)** Human clinical surgical samples and normal adjacent controls; **(B)** Human HNSCC cell lines (FaDu, A-253 and CAL27) and normal human cell lines of head and neck region (HEPM and Hs680.Tr). We observed higher expression levels and activation status of these proteins in tumor samples and HNSCC cells as compared with normal control samples and cell lines. β tubulin served as a loading control. The graphs represent change in protein expression/activation calculated as a ratio (arbitrary units). The results are expressed as means±standard errors of the mean, n=3. α, $p<0.05$; αα, $p<0.01$; ααα, $p<0.001$ represent the statistical significance of protein expression in tumor tissue 1 compared with normal control 1; and β, $p<0.05$ represents the statistical significance of tumor tissue 2 compared with normal control 2; and γ, $p<0.05$; γγ, $p<0.01$; γγγ, $p<0.001$ represent the statistical significance of tumor tissue 3 compared with normal control 3; and δδδ, $p<0.001$ represent the statistical significance of tumor tissue 4 compared with normal control 4 and θθθθ, $p<0.0001$ represent the statistical significance of tumor tissue 5 compared with normal control 5. Similarly, *, $p<0.05$; **, $p<0.01$; ***, $p<0.001$ and ****, $p<0.0001$ represent the statistical significance of protein expression in human HNSCC cell lines compared to HEPM while #, $p<0.05$; ##, $p<0.01$; ###, $p<0.001$ and ####, $p<0.0001$ represent the statistical significance of human HNSCC cell lines compared to Hs680.Tr.

Enlarged images of Fig. 6A in main manuscript:

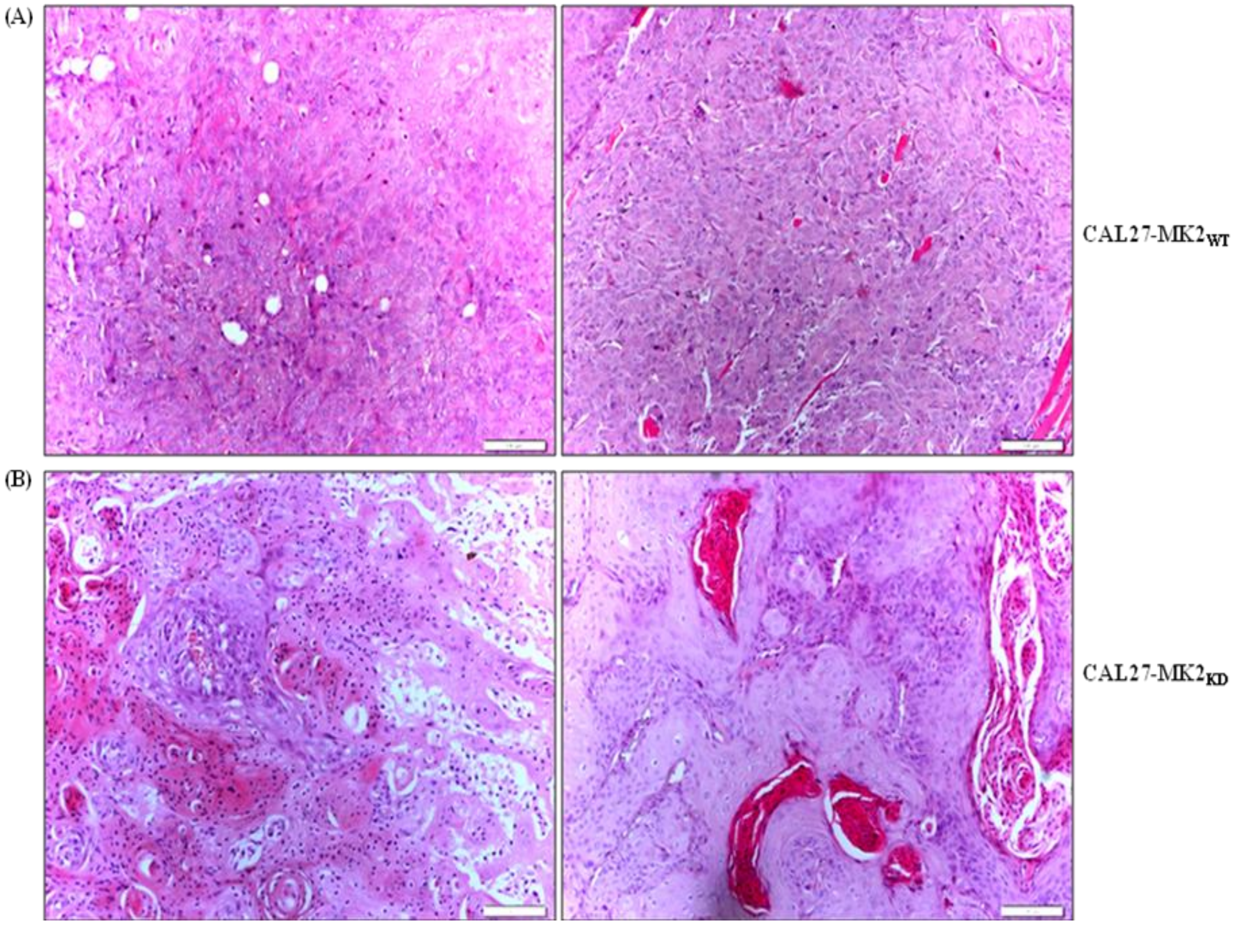

Enlarged images of Fig. 6B in main manuscript:

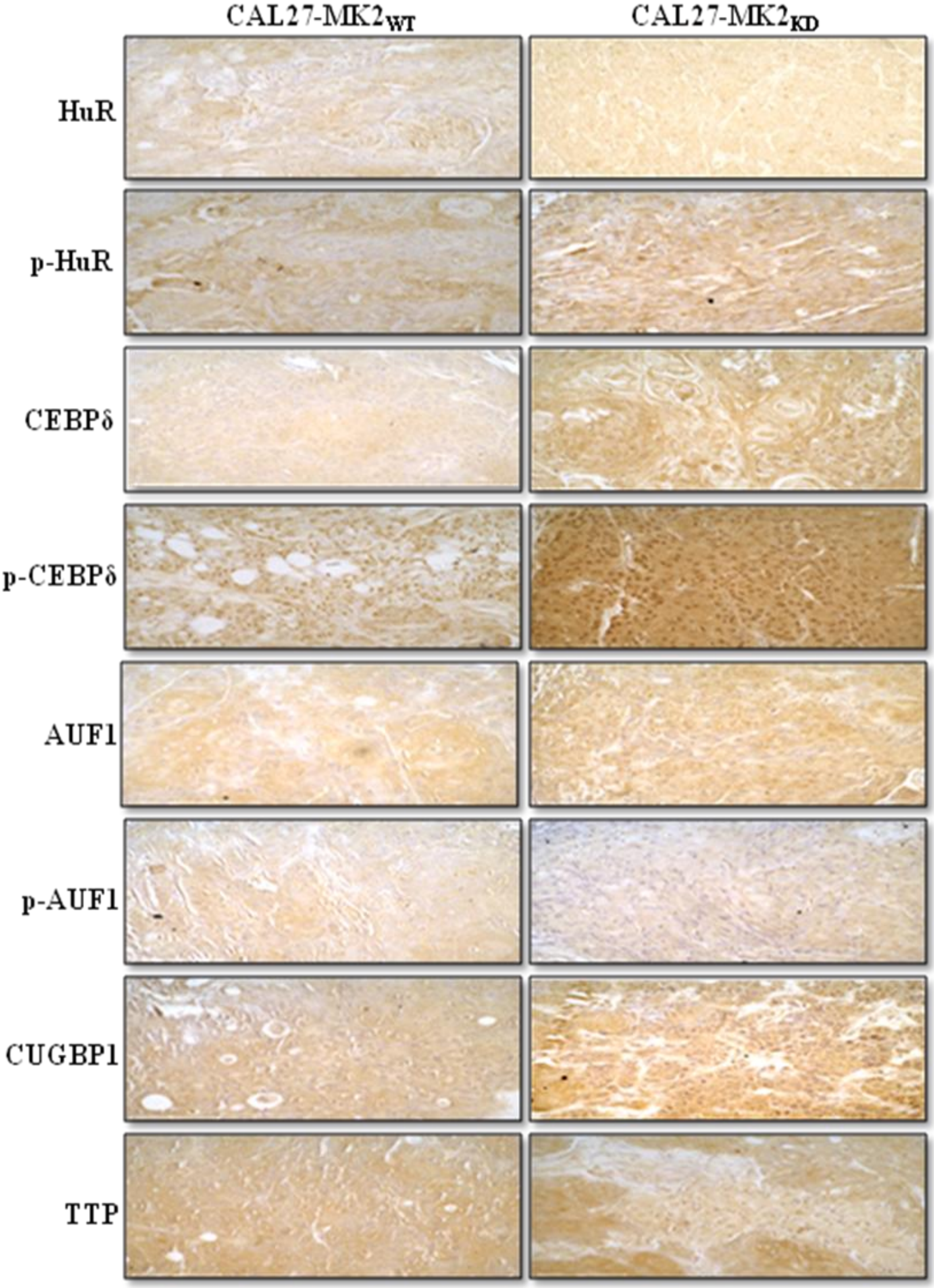

Enlarged images of Fig. 6C in main manuscript:

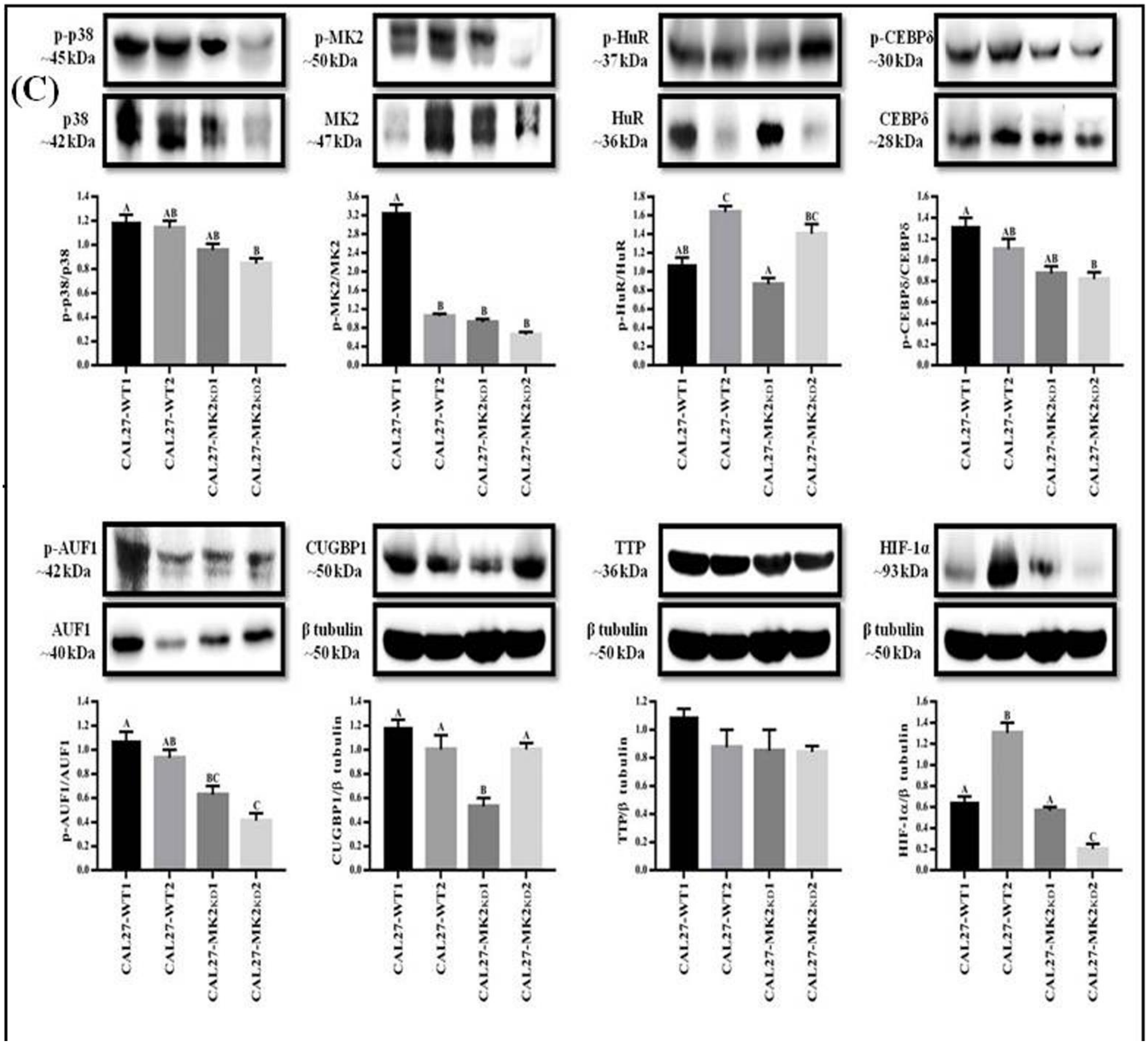

Figure 6: Xenograft establishes that $MK2_{KD}$ attenuates tumor progression. (A) Histopathological examination revealed less differentiated and more aggressive tumors in CAL27-$MK2_{WT}$ than CAL27-$MK2_{KD}$. The images have been captured at 100x and the scale bar denotes 100μm. **(B)** IHC showed expression and activation status of RBPs is more prevalent in CAL27-$MK2_{WT}$ as compared to CAL27-$MK2_{KD}$ group. The images have been captured at 880x (40x objective) and the scale bar denotes 100μm. **(C)** Protein expression analysis using tumor lysates showed that the expression of p38, MK2 and RBPs is higher in CAL27-$MK2_{WT}$ as compared to CAL27-$MK2_{KD}$. β tubulin served as a loading control. The graphs represent change in protein expression/activation calculated as a ratio (arbitrary units). The results are expressed as means±standard errors of the mean, n=3. Significant differences between CAL27-$MK2_{WT}$ and CAL27-$MK2_{KD}$ groups are indicated by different alphabets ($p<0.05$).
